# Experimentally induced REM sleep fragmentation affects psychophysiological habituation to emotional stimuli

**DOI:** 10.1093/sleep/zsaf409

**Published:** 2025-12-23

**Authors:** Lorenzo Viselli, Federico Salfi, Federica Naccarato, Benedetto Arnone, Domenico Corigliano, Giulia Amicucci, Fabiana Festucci, Costanza Colombo, Nicola Cellini, Daniela Tempesta, Michele Ferrara, Aurora D’Atri

**Affiliations:** Department of Biotechnological and Applied Clinical Sciences, University of L’Aquila, Via Vetoio, 67100 L’Aquila, Italy; Department of Biotechnological and Applied Clinical Sciences, University of L’Aquila, Via Vetoio, 67100 L’Aquila, Italy; Department of Biotechnological and Applied Clinical Sciences, University of L’Aquila, Via Vetoio, 67100 L’Aquila, Italy; Department of Biotechnological and Applied Clinical Sciences, University of L’Aquila, Via Vetoio, 67100 L’Aquila, Italy; Department of Biotechnological and Applied Clinical Sciences, University of L’Aquila, Via Vetoio, 67100 L’Aquila, Italy; Department of Psychology, Sapienza University of Rome, Via dei Marsi 78, 00185 Rome, Italy; Department of Biotechnological and Applied Clinical Sciences, University of L’Aquila, Via Vetoio, 67100 L’Aquila, Italy; Department of Biotechnological and Applied Clinical Sciences, University of L’Aquila, Via Vetoio, 67100 L’Aquila, Italy; Department of General Psychology, University of Padova, Via Venezia 8, 35131 Padova, Italy; Department of General Psychology, University of Padova, Via Venezia 8, 35131 Padova, Italy; Department of Biotechnological and Applied Clinical Sciences, University of L’Aquila, Via Vetoio, 67100 L’Aquila, Italy; Department of Biotechnological and Applied Clinical Sciences, University of L’Aquila, Via Vetoio, 67100 L’Aquila, Italy; Department of Biotechnological and Applied Clinical Sciences, University of L’Aquila, Via Vetoio, 67100 L’Aquila, Italy

**Keywords:** REM fragmentation, sleep continuity, emotional memory, emotional reactivity, emotional habituation, emotional processing, skin conductance, heart rate variability, vibrotactile stimulation

## Abstract

Rapid eye movement sleep is believed to reduce physiological reactivity to emotional experiences. While rapid eye movement sleep fragmentation has been associated with maladaptive emotional processing in clinical and animal models, its causal role has not been experimentally isolated in healthy humans. In this study, we tested whether selectively fragmenting rapid eye movement sleep impairs overnight psychophysiological habituation in healthy individuals, aiming to identify the cortical dynamics involved.

Seventeen participants (mean age ± SD, 23.18 ± 3.94, 14 females) completed two counterbalanced conditions (fragmentation and control) each encompassing a baseline assessment of emotional memory/reactivity, a nocturnal polysomnography with or without wrist-applied vibrotactile stimulation during rapid eye movement sleep, a post-sleep emotional memory/reactivity reassessment, and a 48-h follow-up evaluation. Emotional memory was evaluated using an old/new paradigm, while emotional reactivity was assessed through self-report and physiological measures (electrodermal activity and heart rate deceleration–heart rate deceleration).

The stimulation procedure elicited cortical arousal during rapid eye movement sleep, increasing rapid eye movement sleep fragmentation without altering total sleep time, sleep efficiency, and wake after sleep onset. Stimulations reliably induced a distinct cortical arousal signature, characterized by increased higher EEG frequencies (alpha, sigma, beta, gamma). rapid eye movement sleep fragmentation compromised heart rate deceleration habituation to emotional stimuli at both post-sleep assessments without impacting electrodermal response, self-report evaluation, and recognition memory. Crucially, the degree of impaired cardiac habituation at both timepoints was strongly predicted by the magnitude of stimulation-induced alpha power over parieto-occipital regions.

These findings indicated the importance of unperturbed rapid eye movement sleep continuity for proper psychophysiological habituation to emotional events, suggesting alpha intrusions as a potential cortical correlate of impaired habituation.

## Graphical Abstract

**Graphical Abstract:**
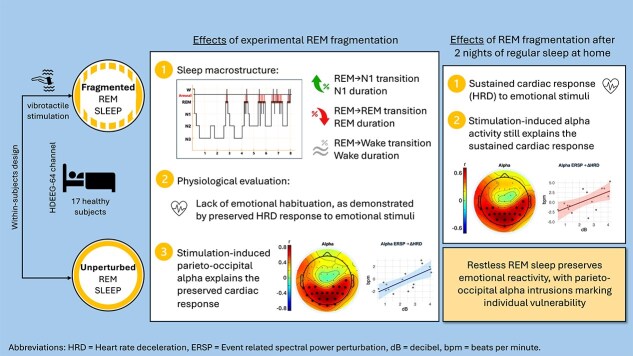

Statement of Significance Fragmented rapid eye movement (REM) sleep is a hallmark of clinical conditions like insomnia, depression, and post-traumatic stress disorder. However, its role in emotional dysregulation in healthy individuals is unclear. This study provides the first experimental evidence that disrupting REM sleep continuity, prompting cortical arousals, impairs the brain’s ability to dampen physiological reactivity to previously encoded emotional events. Interestingly, this lack of emotional reactivity habituation is correlated with parieto-occipital alpha power increases associated with the arousal induction and persists even after regular sleep nights. Our findings establish REM sleep fragmentation as a critical factor for emotional health, suggesting that it is directly involved in emotional dysregulation and may act as a precipitating or maintaining factor in disorders characterized by restless REM sleep.

## Introduction

A key function of rapid eye movement (REM) sleep is the processing of emotional memories [1–3], whose engram comprises both a factual component (the declarative aspect of the experience) and an emotional reactivity component (the intensity of the individual’s affective response to the event). The role of REM sleep in processing the psychophysiological response associated with emotional memory remains a matter of debate, with conflicting evidence deriving from both controlled laboratory studies and more ecological paradigms [2–5].

Previous evidence [6] suggested that total sleep deprivation increases amygdala reactivity to emotional stimuli, reducing its functional connectivity with the medial prefrontal cortex while increasing its connectivity with the locus coeruleus (LC) and the midbrain. Neuroimaging studies further indicated that such amygdala hyperactivation and its altered connectivity with regions involved in top-down emotional regulation are specifically attributable to REM sleep deprivation [7–9]. Moreover, the dampening of emotional reactivity by REM sleep has been negatively associated with frontal gamma (30-40 Hz) power [7], which has been used as a proxy for noradrenergic (NA) tone in mice [10]. Collectively, these findings led to the formulation of the “Sleep to Forget, Sleep to Remember” (SFSR) hypothesis [11], which posits that REM sleep serves a dual function: consolidating the declarative component of the emotional experience while simultaneously dampening the associated emotional reactivity. This differential effect is thought to be possible since elevated levels of acetylcholine, coupled with increased activity in limbic structures (i.e. hippocampus and amygdala), should facilitate emotional memory formation [11]. In contrast, reduced LC activity, leading to the lowest levels of NA, decreases physiological arousal [11]. However, empirical support for a definitive role of REM sleep in emotional processing remains limited.

Partial confirmation of the SFSR hypothesis has been obtained from in vivo studies in animal models, which underscore the importance of reduced NA tone for adaptive emotional processing. Specifically, LC bursts during REM sleep have been found to elevate NA levels, thereby compromising REM continuity [12]. In this context, disrupted REM sleep continuity is operationalized as either an altered temporal clustering of REM bouts (i.e. more frequent, sequentially separated episodes interspersed with brief non-rapid eye movement [NREM] or wake periods) or a reduction in the average duration of individual REM sleep episodes (without altering total REM duration) [13, 14]. Such fragmentation can be experimentally induced by mechanical methods, circadian misalignment or optogenetic activation of the LC [12, 15]. These manipulations introduce NA surges that disrupt REM sleep continuity [12, 16–18], and impair sleep-dependent emotional memory processing in fear conditioning/extinction paradigms [14, 19–21].

In humans, indirect evidence for the role of REM sleep in emotional processing is provided by clinical populations, where distinct yet interrelated conditions such as insomnia, post-traumatic stress disorder (PTSD), depression, and REM sleep behavior disorder are typically characterized by both fragmented REM sleep and maladaptive emotional processing [22–25]. Intriguingly, a recent study on insomniacs by Wassing and colleagues [26] aimed to quantify whether the extent of restless REM sleep was associated with the overnight attenuation of emotional responses showed that overnight amygdala adaptation to emotional experience failed in proportion to the degree of REM sleep discontinuity.

Building on this framework, it has been recently proposed [27] that the silence of NA neurons in the LC during REM would allow the transition of emotional memories from a “novelty” representation, characterized by heightened physiological reactivity, to a “familiar” one, in which reactivity is attenuated. In contrast, phasic bursts of LC activity during REM sleep, triggering sudden NA surges, may hinder emotional habituation by sustaining the novelty status of memory traces, ultimately compromising the regulatory function of REM sleep [27, 28]. As pharmacological and optogenetic manipulations of the LC-NA system directly modulate EEG activation and arousability [17, 18, 29], frequent cortical arousals and sleep stage transitions interrupting REM sleep can be considered an indirect proxy for heightened NA tone in clinical populations, contributing to the emotional dysregulation.

In parallel, recent meta-analyses suggest that REM sleep plays a preferential role in consolidating the declarative component of emotional memories [1, 3]. However, these conclusions are primarily based on a limited number of studies that employ total REM sleep deprivation or split-night paradigms, contrasting the presence of REM sleep with its absence. The question of whether REM sleep fragmentation affects this process is less clear [30]. The perspective proposed by Cabrera and colleagues [27] posits that the strengthening of the factual component of the emotional memory trace occurs primarily during NREM sleep, through the coupling of hippocampal ripples, thalamic spindles and cortical slow oscillations, which have been increasingly implicated in emotional memory processing [27, 31]. This framework suggests a functional dissociation: the processing of the declarative component of the emotional information mostly depends on NREM integrity, whereas REM sleep is more involved in the emotional reactivity dampening. Furthermore, since heightened NA tone, in addition to interfering with long-term depression processes mediating reactivity habituation during REM sleep, may even further promote long-term potentiation in the hippocampus [32–34], REM sleep fragmentation should not specifically affect or compromise the consolidation of the factual component of emotional memory, but could potentially improve it.

Overall, the current findings and theoretical assumptions leave a gap in the literature, as the causal role of the NA tone during REM sleep in emotional habituation has been evaluated only in animal models and clinical populations. Assessing the role of REM sleep fragmentation in healthy subjects, utilizing cortical arousal as a proxy for NA activation in REM sleep, could help in evaluating the role of REM sleep continuity free from confounding factors such as the sleep alterations characterizing sleep in clinical populations.

In this study, we aimed at investigating the effects of REM sleep fragmentation on the ability to consolidate the declarative component of emotional information and to attenuate emotional reactivity, using behavioral, self-report, and physiological measures. We used a sensory stimulation modality designed to induce cortical arousal during REM sleep with low impact on macrostructural sleep parameters, allowing us to study the effect of restless REM sleep on emotional adaptation cleanly. In this regard, we developed a vibrotactile stimulation device consisting of a vibrating bracelet connected to a hardware control system that enabled stimulation management during REM sleep. We hypothesized that experimentally inducing REM sleep fragmentation would compromise emotional habituation, preserving emotional reactivity to previously encoded stimuli. Furthermore, based on the evidence outlined above, we did not expect to find memory impairment due to experimentally induced REM sleep fragmentation.

## Materials and Methods

### Participants

Twenty students were recruited from the University of L’Aquila. Three participants dropped out because they were unable to fall asleep in the laboratory setting. Thus, the final sample comprised 17 subjects (mean age ± SD, 23.18 ± 3.94, age range 19–34, 14 females).

Recruitment criteria encompassed: (1) good sleep quality (Pittsburgh Sleep Quality Index—PSQI [35]—global score < 6; mean ± SD, 3.88 ± 1.17), (2) no insomnia symptoms (Insomnia Severity Index – ISI [36] – score < 7; 2.41 ± 2.24), (3) absence of depression (Depression Anxiety Stress Scale – DASS–21 [37] – depression subscale score < 14; 4.12 ± 3.77), stress (DASS–21 stress subscale score < 19; 9.06 ± 4.42), and anxiety symptoms (DASS–21 anxiety subscale score < 10; 2.12 ± 2.29), (4) regular sleep schedule, (5) normal or corrected-to-normal vision, (6) absence of medication consumption potentially interfering with sleep, (7) absence of sleep disorders, and (8) absence of skin disease, to prevent discomfort during EEG montage or an adverse skin reaction to the conductive gel, and to ensure valid electrodermal recordings. These criteria were assessed during an initial screening using validated questionnaires (PSQI, ISI, DASS-21) and a custom-made questionnaire that inquired about medical history, current medication use, alcohol and other psychoactive substance use, and sleep habits. Female participants were recruited at the end of their menstrual cycle to minimize the influences of hormonal fluctuations on psychophysiological parameters [38, 39].

This investigation was approved by the institutional review board of the University of L’Aquila (protocol no. 49/2021) and was conducted in accordance with the principles outlined in the Declaration of Helsinki.

### Experimental design

**Figure 1:**
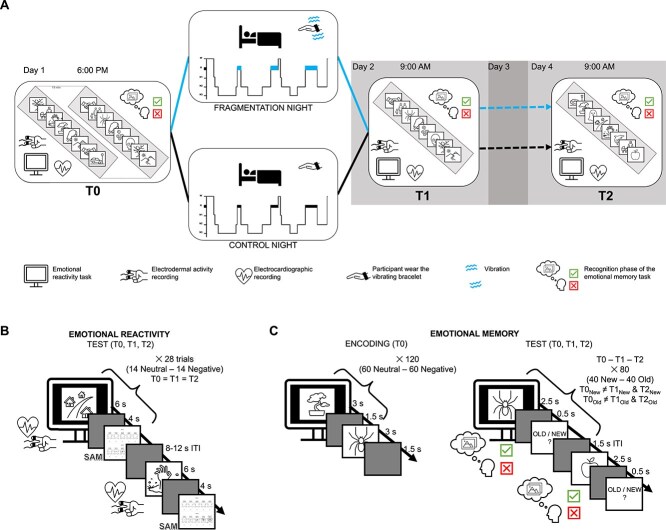
Schematic representation of the experimental protocol and tasks. (A) The study design. On day 1, participants underwent a baseline evaluation of emotional reactivity, followed by stimulus encoding and baseline evaluation of emotional memory performance (T0). Subsequently, they slept in the laboratory undergoing a PSG, either with (fragmentation) or without (control) vibrotactile stimulation. On day 2, participants underwent a new evaluation (T1) of the emotional reactivity and emotional memory in the morning. Finally, on day 4, participants performed a delayed assessment of emotional reactivity and emotional memory performance (T2). (B) The emotional reactivity task. During the presentation of each image, we recorded electrodermal activity and an ECG. After each stimulus, participants rated their perceived valence and arousal using the SAM. (C) The emotional memory task. *Left:* The encoding phase, with the number of stimuli presented for each emotional category. *Right:* The recognition phase, indicating the number of “old” (seen during encoding) and “new” (distractor) images. The set of pictures shown was unique in each test phase.

The participants underwent two experimental conditions (Fragmentation—FRG, Control—CTR; Figure 1A) in a counterbalanced order across subjects, separated by a minimum of 28 days (mean days ± SD, 53.47 ± 36.33) of washout. During the two days preceding the laboratory sleep night, participants’ sleep was monitored at home using an actigraph (see section 3 in Supplementary Materials). On the first experimental day, participants arrived at the laboratory at 5:00 p.m. for the application of electrocardiogram (ECG) and electrodermal activity (EDA) electrodes. The baseline testing session (T0) started at 6:00 p.m. It comprised, in fixed order: 5-min resting-state ECG recording; the emotional reactivity task (~15 min); 5-min post-task resting-state ECG recording; a non-emotional distractor task (~15 min); the stimulus encoding phase of the memory task (~10 min) and, after a 10-min break, the immediate recognition test (~8 min). Following a dinner break, participants were prepared for the polysomnography (PSG). Bedtimes were scheduled between 10:30 p.m. and 12:30 a.m., according to each participant’s habitual bedtime. Participants were allowed to sleep for 8 h, timed from the first epoch of NREM stage 2 sleep. If no final awakening spontaneously occurred within this timeframe, the final awakening was scheduled with ±20-min flexibility to avoid disrupting an ongoing REM sleep period or the fragmentation procedure. Approximately 1 h after the final awakening (between 08:00 a.m. and 10:00 a.m.), the post-sleep test session (T1) started and included: the 5-min resting-state ECG recording, followed by the emotional reactivity task (~15 min) and the post-task 5-min resting-state ECG recording; the non-emotional distractor task (5 min); the recognition test phase of the emotional memory task (~8 min). Participants left the laboratory after the T1 session, and their sleep during the next two nights was monitored via actigraphy. 48 h after the T1 testing phase, participants returned to the laboratory (arrival time scheduled according to the T1 start time) for the delayed test session (T2), which replicated the same sequence of activities as described for T1.

### REM sleep fragmentation procedure

The vibrotactile stimulation device was a novel apparatus developed in-house specifically for this study. Its conceptual design was directly inspired by previous work demonstrating that vibrotactile stimuli applied to the arm can effectively induce cortical arousals during sleep [40, 41]. At the same time, similar principles of sensory stimulation have been employed during sleep in recent research for different purposes [42]. The stimulation device comprised a vibrating bracelet connected to a hardware control system. The vibrating bracelet consists of an elastic fabric wristband housing five 5 V vibration motors. The hardware control system comprises an Arduino Uno Rev3 board (ARDUINO, Italy), a printed circuit board, a 5 V Micro SD card module for Arduino, a 5 V relay, and a power regulator. All these hardware components were enclosed in an ABS plastic module case and powered by an external source.

The stimulation device operated in a semi-automated manner. Upon activation, it generated a continuous automated stimulation-pause cycle, consisting of a 3-s vibration followed by a 3-s pause. This stimulation-pause cycle could be suspended for 40 s or terminated, depending on the specific user input to the control system.

Vibrotactile stimulations were managed by a sleep expert, who continuously monitored the EEG recording throughout the night to fragment REM sleep. The semi-automated stimulation-pause cycle was applied during all REM sleep periods throughout the night and initiated once the first REM sleep epoch, according to AASM criteria [43], was identified.

The stimulation procedure followed a strict, rule-based scheme. The vibration intensity was manually controlled via a 7-step graduated knob that adjusted the operating voltage (V) of the motors within a 0.8–4.5 V range. At the beginning of each new REM period, the procedure was always initiated at the minimum intensity (0.8 V). Each stimulation lasted up to 3 s or was terminated sooner if cortical arousal appeared on any EEG trace. In the absence of a cortical response to the stimulation, the sleep expert manually increased the vibration intensity by one pre-calibrated step during the 3-s pause between stimuli until a clear EEG arousal was observed. If the participant remained in REM sleep after this arousal, the sleep expert paused the stimulation, which automatically restarted after 40 s with the previous effective intensity. However, if the stimulation caused a sleep stage transition or body movement, it was stopped until the participant returned to REM sleep. Upon the participant’s return to REM sleep, the stimulation procedure was re-initiated from the minimum intensity level.

### Experimental tasks

We developed the tasks with PsychoPy (v2020.2.10) and presented them on a 24-inch monitor (NILOX, NXMMIPS240004) powered by a Mac mini (Apple M1, 2020).

### Emotional reactivity task

The emotional reactivity task assessed both subjective and psychophysiological responses to emotional stimuli and evaluated potential differences in resting-state heart rate variability (HRV) parameters. The task (Figure 1B) encompassed 5 min of resting-state ECG recording before and after performing 28 trial each involving: (1) emotional picture presentation (14 emotionally negative, 14 emotionally neutral) for 6 s, (2) 4 s interstimulus interval, (3) subjective rating of image arousal and valence on a 9-point Likert scale utilizing the self-assessment manikin (SAM) [^44^], and (v) a variable intertrial interval (ITI) ranging from 8 to 12 s, which was jittered to prevent anticipatory skin conductance response (SCR). Participants were given unlimited time for each rating to avoid time–pressure effects on their emotional evaluation and to allow for minor postural adjustments between trials. A total of 28 negative and twenty-eight neutral images were selected to create four distinct sets of images, thereby differentiating stimuli between conditions. The negative stimuli consisted of the most arousing and gruesome images from the International Affective Picture System (IAPS) [45], while the neutral ones comprised white background images of sports objects from the Mnemonic Similarity Task [46] (see section 1 in the Supplementary Material for details on the stimuli).

### Emotional memory task

The emotional memory task assessed the declarative component of emotional information. This task (Figure 1C) consisted of a stimulus-encoding phase, followed by a recognition test at each session (T0, T1, T2). The encoding phase consisted of presenting 120 emotional pictures (60 negative and 60 neutral) individually, displayed for 3 s each with a 1.5-s interstimulus interval during which a black screen was shown. Participants were required to memorize the stimuli presented during the encoding for the subsequent recognition phases. In each recognition phase, participants were requested to discriminate between the stimuli presented during the encoding phase (i.e. “OLD”) and new pictures (i.e. “NEW”). Each recognition test phase consisted of presenting 80 emotional pictures, half of which were completely new (20 negative and 20 neutral), while the other half were taken from the encoding phase set (20 negative and 20 neutral). The NEW and OLD images differed during each test phase. In each recognition phase, emotional pictures were displayed for 2.5 s and, after a 0.5 s, participants had to answer whether the picture was OLD or NEW (participants were given unlimited time to make their judgment to prioritize recognition accuracy over response speed), then a fixation cross was displayed during the 1.5 s ITI. To develop the task, a total of 480 images (240 negative, 240 neutral) were selected from the IAPS and the Nencki Affective Picture System (NAPS) [45, 47] (see section 2 of the Supplementary Material for the stimulus details).

### Data acquisition and pre-processing

#### Actigraphic monitoring of sleep at home

Participants’ sleep at home during the two nights preceding T0 and following T1 was monitored via actigraphy (GENEActiv accelerometer—Activinsights Ltd., Kimbolton, UK) to control for initial comparability of the experimental conditions at baseline and to evaluate possible effects of REM sleep fragmentation on subsequent sleep (see section 3 of the Supplementary Material for details on data pre-processing and statistical analyses).

#### Polysomnography

PSG raw data were acquired using the BrainVision Recorder (Version 1.26.0101, Brain Products GmbH, Germany). The setup included 64-channel EEG, electrooculogram (EOG), electromyogram (EMG), and ECG signals. The onset/offset of the vibrotactile stimulations during the FRG night were marked on the PSG recording via the Trigger-Box Plus (Brain Products GmbH, Germany), which connected the stimulation device to the EEG acquisition system.

EEG and EOG signals were recorded with a BrainCap connected to a BrainAmp MR amplifier (Brain Products GmbH, Germany) with a sampling rate of 500 Hz, high-pass filtering at 0.016 Hz, and applying a 50 Hz notch filter. EMG and ECG signals were acquired with Ag/AgCl electrodes via a BrainAmp ExG MR with a sampling rate of 500 Hz. The EMG signal was high-pass filtered at 10 Hz, while the ECG was high-pass filtered at 0.001 Hz; a 50 Hz notch was applied for both signals. Impedances were kept below 5 kΩ for EEG and EOG, 10 kΩ for EMG, and 15 kΩ for ECG signals.

Sleep staging was performed manually by a sleep expert according to AASM criteria [43] using the Python-based Wonambi package (v7.11, https://wonambi-python.github.io/index.html) [48] to visualize the PSG recording. Then, we derived the following variables: (1) Total Sleep Time (TST, min), representing the sum of time spent in N1, N2, N3, and REM sleep; (2) Sleep Onset Latency (SOL, min), calculated as the time elapsed between the moment the participant was in bed ready to attempt to fall asleep (signaled via five consecutive eye blinks) and the first sleep epoch (N1 or N2); Wake After Sleep Onset (WASO, min); (3) Sleep Efficiency (SE, %) as TST/Time in bed (TIB, min) × 100; (4) sleep stages duration in minutes and as a percentage of TST; (5) REM latency, indicating the time elapsed between the sleep onset and the first REM sleep epoch; (6) #awakenings, denoting the total number of awakenings during the sleep period; (7) REM sleep fragmentation index (REMfr) computed as the total number of cortical arousals, body movements and bouts of NREM sleep and wakefulness that interrupted REM sleep, divided by the total duration of REM sleep in hours.

Furthermore, PSG-derived sleep macrostructure was analysed using a Markov transition matrix to evaluate whether sleep continuity, operationalized as the probability of transitions between sleep stages (including wakefulness), differed due to the vibrotactile stimulation paradigm. Markovian transition probabilities *P_ij_* were computed for each pair of sleep stages as the conditional probability of an epoch being in stage *j*, given that the preceding epoch was in stage *i*. Mathematically, *P_ij_* was estimated as the ratio of epochs in which stage *i* was immediately followed by stage *j*. A 5 × 5 transition matrix for participants was derived, and we then calculated the mean and standard deviation for each sleep stage transition in both conditions.

We then investigated the EEG responses to nocturnal tactile stimulation using EEGLAB (v2023.0) [49]. The continuous EEG signal was first band-pass filtered between 0.3 and 45 Hz. Then, the REM sleep periods were visually inspected to identify and interpolate any problematic channels. Subsequently, we extracted epochs time-locked to the onset of each stimulation, spanning from –3000 ms to 15 000 ms. From this set of epochs, we applied a strict selection process. First, we discarded all epochs in which stimulation occurred outside REM sleep (mean ± SD, 1.65 ± 2.26). Second, from the stimulation-pause sequences, we retained only the epoch corresponding to the final stimulation of each train, as this was the one that elicited a cortical activation. Third, we rejected any epochs containing body movements or other artifacts that could not be corrected via interpolation. Finally, we also discarded any epoch in which stimulation led to a full awakening (i.e. a transition to stage W), to isolate the specific effects of cortical arousals during sleep. A time-frequency representation of the selected epochs was then computed using the “*newtimef*” function in the EEGLAB toolbox to evaluate the spectral correlates of the final vibrotactile stimulation. Each epoch was convolved with a complex Morlet wavelet, spanning frequencies from 5 to 40 Hz, with a frequency resolution of 0.2 Hz and a time step of 16 ms. The lower frequency bound was set at 5 Hz to mitigate potential low-frequency artifacts from rapid eye movements in REM sleep [50] To balance the trade-off between temporal and frequency resolution, we used an adaptive number of wavelet cycles that increased from 7 cycles at the lowest frequency (5 Hz) to 42 cycles at the highest frequency (40 Hz). This approach was chosen to deliberately favor frequency precision, allowing for a clear distinction of the stimulation’s impact across different frequency bands. For each subject and EEG channel, the event-related spectral perturbation (ERSP) was calculated by averaging the time-frequency representations across all selected epochs. The resulting spectral power values at each time-frequency point were then baseline-corrected by dividing them by the mean spectral power at the same frequency within a pre-stimulus time window from –2000 to –1000 ms, which, in the case of stimulation sequences, fell within the mandatory 3-s pause between the stimulation events in a train. Finally, these ERSP values were converted to a decibel (dB) scale [dB = 10^*^log10(power/baseline)].

### Objective emotional reactivity: EDA and heart rate

EDA and ECG signals were acquired using the Biosignal Explorer (Biosignalsplux, PLUX wireless biosignals S.A., Lisbon, Portugal) with a sampling rate of 1000 Hz and a resolution of 16 bits.

EDA raw data were handled in MATLAB (R2024b, Update 5, 24.2.0.2863752, The MathWorks Inc., Natick, Massachusetts) employing Ledalab [51]. The signal was pre-processed by applying a 10 Hz down-sampling, a 1 Hz second-order low-pass Butterworth filter, and a smoothing with a Gaussian window width of 10 data points (1 s). Then, a trial-by-trial visual inspection was conducted to reject artifacts based on their morphology (e.g. abrupt steep-slope spikes, characteristic of movement) [52], resulting in the exclusion of an average of 2.77 (±2.59) trials per participant (1.65% ±1.54%). Subsequently, we selected an SCR amplitude threshold of 0.05 μS within a response window of 1 to 6 s after stimulus onset [53], before performing a continuous decomposition analysis (CDA) [51]. From the CDA, we derived the *CDA.SCR* index representing the average phasic activity detected within the response window [51].

ECG raw data were processed in Artiifact [54], to extract the interbeat intervals (IBIs). IBIs data were processed using the Berntson detection method to identify artifacts, and cubic spline interpolation was adopted as the correction method. From these artifact-corrected IBIs in the resting-state ECG recordings, we extracted different HRV indices (see section 4 in the Supplementary Materials for details). Artifact-corrected IBIs derived from ECG raw data of the emotional reactivity task were processed in MATLAB using Kardia [55] to extract the phasic cardiac responses to stimulus onset, allowing for the calculation of heart rate deceleration (HRD). HRD was computed by subtracting the lowest heartbeat value collected during the 6 s post-stimulus onset from the mean heartbeat recorded in the 2 s before stimulus presentation. The HRD is an attentional orienting response that reflects stimulus elaboration, with higher HRD levels associated with the processing of emotionally salient information [56, 57].

### Subjective emotional reactivity

Participants provided their arousal and valence ratings during the emotional reactivity task on a Likert scale ranging from 1 to 9 using the numeric keypad, based on the SAM [44]. The SAM scale depicted a cartoon-type manikin representing human emotional expressions, ranging from smiling and happy to frowning and unhappy for the valence rating, and from calm and relaxed to excited and wide-eyed for the arousal evaluation. We derived *Valence* and *Arousal* variables to assess subjective emotional reactivity.

### Emotional memory performance

Emotional memory performance was evaluated by computing the d-prime (*d′*). The d′ is a measure of sensitivity, reflecting the ability to discriminate a target stimulus (OLD picture) from a non-target stimulus (NEW image), and is unaffected by response bias [58]. Higher d′ values denote finer discrimination ability.

We derived the d′ by calculating the Hit Rate and the False Alarm Rate. The Hit Rate indicates the number of hits (i.e. the OLD pictures correctly identified as seen) divided by the total count of OLD pictures in the recognition task, specifically *Hit Rate = Hits / N_OLD_*. The False Alarm Rate indicates the False Alarm (i.e. the number of NEW images erroneously defined as seen) divided by the total count of NEW images in the recognition task, namely False Alarm Rate = False Alarm / N_NEW_. Then, to calculate the sensitivity index d′, the Hit Rate and False Alarm Rate values for each participant were converted to their corresponding z scores using the inverse of the standard normal cumulative distribution function. The d′ was then computed by applying the formula: *d′ = z_HitRate_ – z_FalseAlarmRate_*. Since d′ must not be computed when Hit Rate = 1 and False Alarm Rate = 0, we replaced Hit Rate values of 1 with a *Hit Rate = 1 – 1*/*(2 N)* and False Alarm Rate values of 0 with a *False Alarm Rate = 1/2 N* (i.e. N indicates the number of targets) [58].

### Statistical analyses

#### Effects of writs-applied vibrotactile stimulation on sleep macrostructure

A paired-sample *t*-test (Students’ *t*) was performed to compare PSG sleep parameters between the CTR and FRG conditions, evaluating whether the vibrotactile stimulation paradigm altered participants’ nocturnal sleep. Paired *t*-tests on PSG sleep variables were conducted without correction for multiple comparisons, as the goal was to verify the absence of significant differences between conditions. Not applying corrections reduced the risk of inflating Type II error, thus offering a more stringent test of equivalence.

To assess differences in sleep continuity, the transition probabilities between sleep stages, derived from the Markovian transition matrices, were compared between conditions. A paired *t-*test was performed for each corresponding cell of the matrices, and the resulting statistical contrasts were corrected for multiple comparisons using the Bonferroni method.

#### E‌EG correlates of vibrotactile stimulation during REM sleep

To identify significant spectral perturbations induced by the stimulation at the single-channel level, we performed a statistical analysis using the FieldTrip toolbox [59]. A paired-samples *t*-test was conducted, comparing each time-frequency point in the 14-s post-stimulus window against the mean power of the corresponding frequency bin from the baseline period (the mean of the –2000 to –1000 ms window). To correct for multiple comparisons across time and frequency points, we applied a cluster-based permutation test using a Monte Carlo method with 5000 random permutations. Next, to investigate the topographical distribution of these spectral changes, we calculated the mean ERSP within distinct frequency bands: theta (5–7.80 Hz), alpha (8–11.80 Hz), sigma (12–15.80 Hz), beta (16–29.80 Hz), and low-gamma (30–40 Hz) in the 6 s post-stimulus window. For each channel and frequency band, the resulting mean ERSP value was compared with its respective baseline value using a paired-samples *t*-test. Again, a cluster-based Monte Carlo method (5000 permutations) was applied to correct for multiple comparisons across the scalp topography. For all analyses, the significance threshold for cluster formation (cluster-alpha) was set at *p* < .05, and the overall alpha level for determining cluster significance was also *p* < .05, two-tailed.

#### Effects of REM sleep fragmentation on emotional memory and emotional reactivity

Distinct linear mixed models (LMMs) were employed to identify potential differences in emotional reactivity indices and memory performance between the CTR and FRG conditions. The models embedded, as dependent variables: *Valence* and *Arousal* ratings for subjective emotional reactivity; *CDA.SCR* and *HRD* for objective emotional reactivity, and the *d′* for memory performance. Each LMM model comprised the factors *Condition* (CTR, FRG), *Session* (T0, T1, T2), *Stimulus Type* (Negative, Neutral), and their interaction as predictors.

For each model, the participant was entered as a cluster variable, and a random intercept was included per participant, accounting for intraindividual variability and measure-correlation among participants. In the models performed on the emotional reactivity indices, the stimulus ID was included as a cluster variable, and a random intercept was placed for the stimulus ID, considering the use of identical stimuli between sessions in each condition.

For LMMs analyses, the interpretation of significant effects followed a hierarchical approach; significant main effects were subordinated to the absence of significant interaction effects involving the same factor, and the interpretation of interaction effects was subordinated to the lack of significant higher-order interaction effects. Furthermore, given the extensive nature of the analyses, our reporting strategy prioritizes the main factor of interest: *Condition*. Therefore, only significant main effects or interactions involving the *Condition* factor are detailed in the main manuscript. Any other significant effects (e.g. main effects of *Session* or *Stimulus Type* not interacting with *Condition*) are reported in the Supplementary Materials.

Considering the study aims, we performed Bonferroni-corrected planned comparisons for significant interaction effects to reduce the risk of Type II errors when correcting for multiple comparisons. Specifically, we investigated possible differences between sessions in each condition (i.e. T0 vs T1, T0 vs T2, and T1 vs T2) to determine how our variable of interest evolved in the CTR and FRG conditions. Moreover, we compared CTR and FRG conditions at each time point, examining potential baseline differences (i.e. CTR at T0 vs FGR at T0) and evaluating short-term (i.e. CTR at T1 vs FGR at T1) and long-term effects (i.e. CTR at T2 vs FGR at T2).

Finally, to directly link the possible effects of REM sleep fragmentation on emotional memory and reactivity to the effects of nocturnal stimulation on sleep EEG, we performed Pearson’s correlation analyses. We correlated the over-session changes in our primary behavioral and physiological variables of interest (calculated as the delayed test score minus the baseline test score, for both T1 and T2) with the mean ERSP at each channel for each frequency band within the 6 s post-stimulus window. To correct for multiple comparisons across the scalp topography, a cluster-based Monte Carlo permutation test (5000 permutations) was applied to the correlation results. For graphical representation purposes, in the case of a significant cluster, the mean ERSP value was extracted from all channels within that cluster and was then correlated with the over-session changes in the behavioral variable. This allowed for a clear visualization of the significant relationship between electrophysiological and behavioral variables.

All the above-reported analyses were performed in Jamovi (Version 2.6, The Jamovi Project, Sydney, Australia) and MATLAB (R2024b, Update 5, 24.2.0.2863752, The MathWorks Inc., Natick, Massachusetts). All tests were two-tailed, and statistical significance was set at *p* < .05.

## Results

### Control analysis of actigraphic-recorded sleep preceding the experimental sessions

No significant difference in the sleep macrostructure of the two nights preceding the participation emerged from the comparisons between FRG and CTR conditions (see section 3.3 and Tables S5 and S6 in the Supplementary Materials for details), confirming the comparability of the sleep features.

### Effects of vibrotactile stimulation on REM sleep continuity and macrostructural sleep parameters

Wrist-applied vibrotactile stimulation has been effective in interrupting REM sleep continuity as reflected by a higher *REMfr* in the FRG condition (Table 1). Experimentally induced REM sleep fragmentation resulted in reduced REM sleep duration (mean difference = -22.94 min) and percentage (mean difference = -5.13 %), accompanied by increased N1 duration (mean difference = 20.12 min) and percentage (mean difference = 4.71 %) (Table 1). No other sleep parameters were affected by the experimentally induced REM sleep fragmentation.

**Table 1:** Mean ± SD of PSG sleep parameters in each condition and their statistical comparisons

Variable	FRG	CTR	t_16_	p
TST	430.59 ± 26.03	433.68 ± 30.27	-0.51	0.620
SOL	17.29 ± 11.20	16.32 ± 12.03	0.37	0.715
WASO	43.21 ± 22.61	53.03 ± 33.69	-1.53	0.146
SE %	87.77 ± 4.22	86.53 ± 7.22	0.88	0.390
**N1 (min)**	**57.32 ± 14.68**	**37.21 ± 7.31**	**5.75**	**<0.001**
N2 (min)	217.35 ± 27.25	216.53 ± 33.32	0.14	0.887
N3 (min)	92.97 ± 23.58	94.06 ± 22.96	-0.32	0.752
**REM (min)**	**62.94 ± 8.13**	**85.88 ± 19.44**	**-5.65**	**<0.001**
**N1 %**	**13.37 ± 3.54**	**8.66 ± 1.94**	**5.84**	**<0.001**
N2 %	50.39 ± 4.84	49.83 ± 6.23	0.50	0.626
N3 %	21.63 ± 5.42	21.77 ± 5.30	-0.16	0.872
**REM %**	**14.61 ± 1.62**	**19.75 ± 4.01**	**-6.16**	**<0.001**
REM latency	126.82 ± 40.91	135.76 ± 59.73	-0.56	0.584
#awakenings	21.71 ± 9.69	19.88 ± 5.79	0.98	0.341
**REMfr**	**75.52 ± 10.03**	**23.12 ± 5.43**	**23.29**	**<0.001**

**Abbreviations:** TST = total sleep time, SOL = sleep onset time, WASO = wake after sleep onset, SE = sleep efficiency, REM = rapid eye movements sleep, REMfr = REM sleep fragmentation index. Significant comparisons are reported in bold.

**Figure 2:**
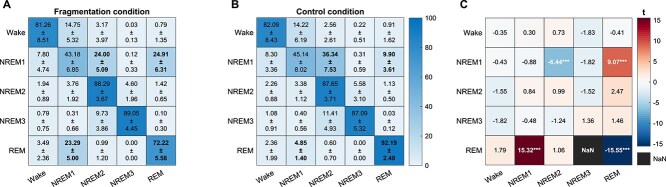
Markovian matrices of sleep stage transition probabilities. (A and B) Mean ± SD transition probabilities (%) between sleep stages for the (A) control (CTR) and (B) fragmentation (FRG) conditions. In each matrix, the rows represent the originating stage, and the columns represent the destination stage. Values corresponding to statistically significant differences (shown in C) are highlighted in bold. (C) Matrix of t-values from a paired-samples t-test comparing transition probabilities between conditions. Positive t-values (red scale) indicate a higher transition probability in the FGR condition, while negative t-values (blue scale) indicate a higher probability in the CTR condition. NaN indicates transitions where the *t*-value could not be computed. Asterisks denote significant differences; ^***^p < .001.

The statistical comparisons of the Markovian transition matrix (Figure 2) revealed a decreased REM sleep continuity in the FRG condition, as indicated by a lower probability of REM sleep being followed by another REM episode. Indeed, the transition from REM to N1 increased. At the same time, N1 had a reduced probability of being followed by N2. The REM sleep manipulation technique did not increase the probability of transitions to Wakefulness.

### E‌EG response to vibrotactile stimulation during REM sleep

**Figure 3:**
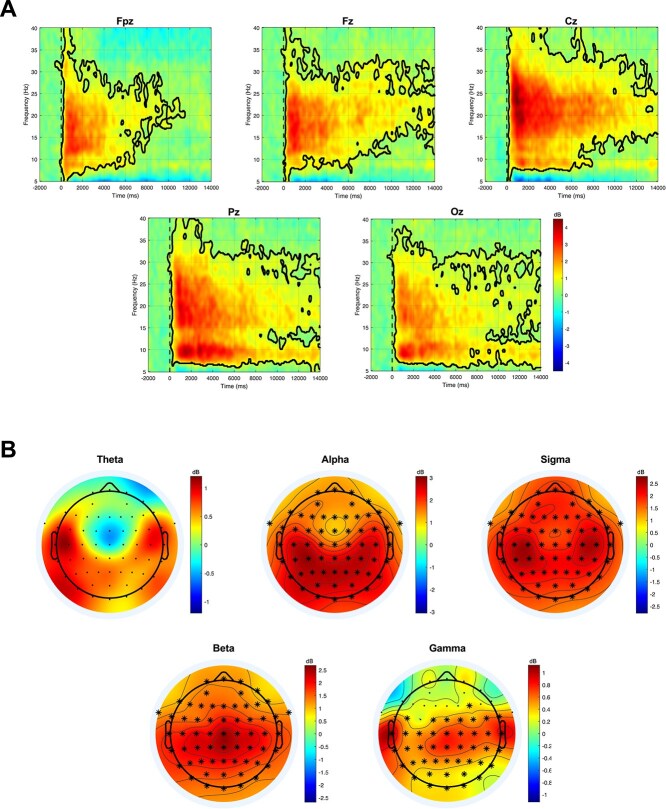
Stimulation-induced changes in EEG spectral power. (A) Time-frequency representation of the ERSP for the five representative midline electrodes. The color bar indicates the power changes in dB relative to the pre-stimulus baseline period. The black contour lines enclose time-frequency regions where the spectral power change was statistically significant (*p* < .05, cluster-corrected). (B) Topographical distribution of the mean ERSP within five distinct frequency bands, averaged over the first 6 s post-stimulus window. The color scale represents the magnitude of the ERSP difference from baseline. Electrodes that are part of a significant positive (red) cluster are highlighted with an asterisk (^*^, *p* < .05, cluster-corrected).

The statistical analysis of the ERSP revealed significant changes in neural oscillatory activity following the vibrotactile stimulation. Figure 3A illustrates the time-frequency dynamics of these changes for five representative midline electrodes (Fpz, Fz, Cz, Pz, Oz). These electrodes were selected to provide a clear and concise representation of the stimulation effects across the anteroposterior axis, avoiding the redundancy of presenting plots for all channels. The results showed a significant increase in power across a frequency range spanning approximately 8 Hz to 40 Hz. This power increase was observed across all considered midline derivations (Fpz, Fz, Cz, Pz, and Oz) and persisted for at least ~6 s at frequencies below 30 Hz. The increase was more transient for frequencies above 30 Hz, where the activity returned to the baseline level within the first few seconds following the stimulation.

To visualize the spatial distribution of these spectral changes, we analysed the topographical maps of the mean ERSP for each frequency band in the 6-s post-stimulus window (Figure 3B). The analysis confirmed the patterns observed in the time-frequency plots. The alpha, sigma, and beta bands exhibited a widespread and significant power increase that extended across the entire scalp. The low-gamma band also showed a significant power increase, though this effect was predominantly localized to centro-parieto-occipital regions.

### Effects of REM sleep fragmentation on emotional memory

The LMM on *d′* indicated no significant main effect of the *Condition* factor or any interaction involving it (all *p* ≥ .141). Regarding the other factors and their interactions, we observed a significant main effect of the *Session* factor, indicating that emotional memory performance decreased over time, regardless of the experimental condition and the emotional valence of the stimuli (see section 5 in the Supplementary Materials for details).

### Effects of REM sleep fragmentation on emotional reactivity habituation

The LMM performed on *Arousal* ratings revealed no significant effects involving the *Condition* factor (all *p* ≥ .083). In contrast, a significant main effect of the *Stimulus Type* factor emerged, with negative pictures perceived as more arousing than neutral pictures (see section 6 and Figure S2 in the Supplementary Materials for details). No other main effects or interactions reached statistical significance.

For *Valence* ratings, the LMM showed a significant *Condition × Stimulus Type* interaction (F_(1,2791.37)_ = 9.23, *p* = .002). Planned Bonferroni comparison revealed that negative images were rated as less pleasant than neutral images in both the CTR (mean difference = 3.50, t = 35.81, *p*<.001) and FRG (mean difference = 3.74, t = 38.26, *p*<.001) conditions. Moreover, neutral images were rated as more pleasant in the FRG condition relative to the CTR condition (mean difference = 0.29, t = 5.26, *p*<.001) (see section 6 and Figure S3 in the Supplementary Materials).

EDA and ECG data from the emotional reactivity task for two participants were excluded from the analyses because one participant failed to follow the instruction to limit movement to reduce recording artifacts, and the other had missing data due to a recording failure. Moreover, two participants were categorized as non-responders [60, 61] and excluded from the EDA analysis as they showed a response to fewer than 20% of negative stimuli during at least one baseline (T0) session (a criterion adapted from Lonsdorf and colleagues) [61]. Thus, the LMM models for *HRD* and *CDA.SCR* were based on fifteen and thirteen participants, respectively.

The LMM analyses on *CDA.SCR* highlighted no significant effects involving the *Condition* factor, nor any significant interaction involving it (all *p* > .069). We found a significant *Session × Stimulus Type* interaction, indicating a habituation effect to negative stimuli over time (see section 6 in the Supplementary Material for details). No other comparisons yielded significant differences (all *p*≥.999).

**Figure 4:**
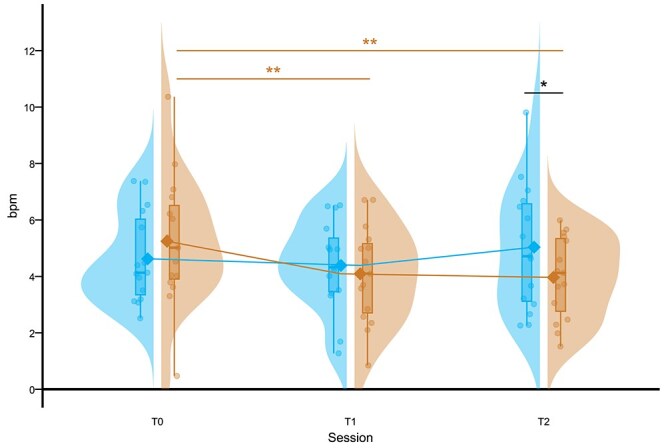
Effects of REM sleep fragmentation on *HRD*. The raincloud illustrates the *HRD* values in beats per minute (bpm) across the session (T0, T1, T2) for the CTR (copper) and FRG (light blue) conditions. Each half of the visualization includes: A split violin plot, showing the probability density of the data distribution; an embedded boxplot, displaying the median (horizontal line) and the interquartile range (the box); individual data points, representing the mean *HRD* for each participant. The solid lines and the diamond-shaped markers connect the estimated marginal means from LMM for each condition. The horizontal bars indicate significant pairwise Bonferroni-corrected planned comparisons: the copper-colored bars show significant changes within the CTR condition, while the black bar indicates a significant difference between conditions (^**^  *p* < .01; ^***^  *p* < .001).

Regarding the *HRD* index, the LMM analyses highlighted a significant *Condition × Session* interaction (F_(2,2408.17)_ = 6.12, *p* = .002), indicating that the *HRD* trajectory over time differed between the two groups (Figure 4). Bonferroni-corrected planned comparisons indicated two distinct response patterns: in the CTR condition, *HRD* exhibited a clear habituation-like response, decreasing from T0 to T1 (t = 3.36, *p* = .007) and remaining stable at T2 (t = 0.37, *p* > .999). Conversely, in the FRG condition, the *HRD* remained stable across all sessions, with no significant changes observed over time (all *p* > .529). Finally, *HRD* was higher at T2 during the FRG condition than in the CTR condition (t = 3.12, *p* = .016) (Figure 4). This divergence in HRD trajectories suggests that REM sleep fragmentation interfere with the overnight emotional habituation.

No significant differences in resting-state HRV emerged between the FRG and CTR conditions before or after the emotional reactivity task (see section 4 in the Supplementary Materials for details).

### Relationship between induced spectral power perturbation during REM sleep fragmentation and changes in psychophysiological reactivity

**Figure 5:**
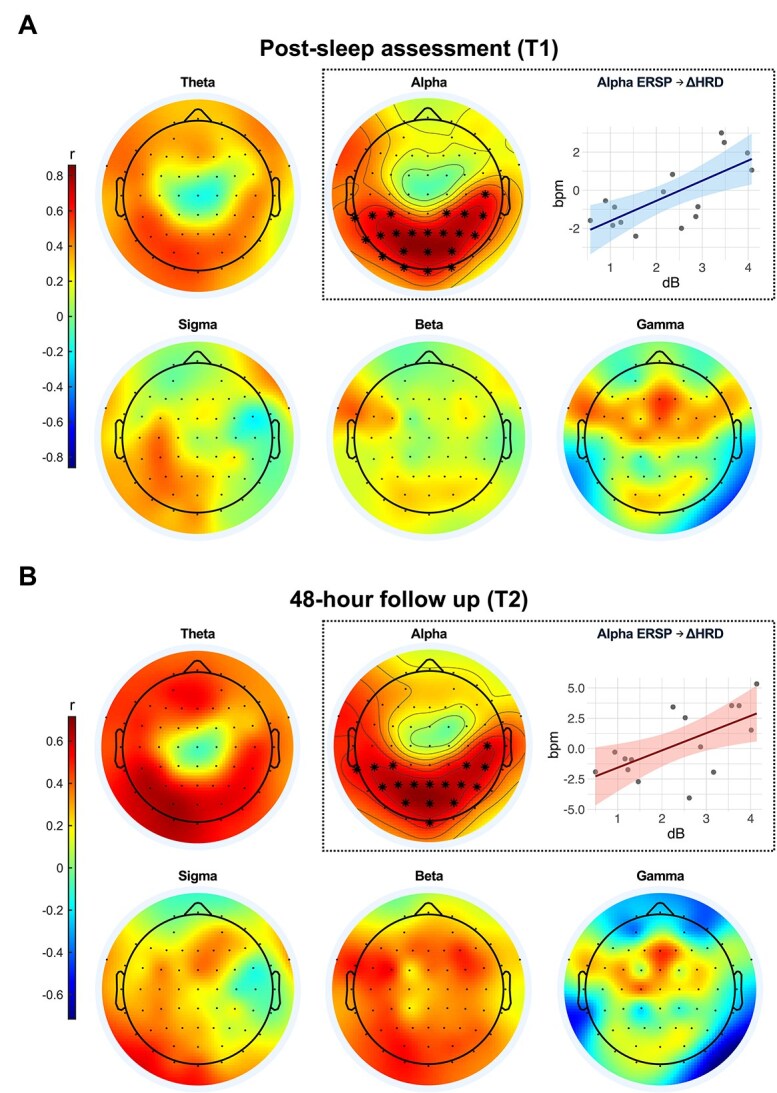
Pearson’s correlation between vibration-induced spectral power perturbations and over-session changes in *HRD* (*ΔHRD*). (A) Correlation analysis for the change in *HRD* from baseline to the T1 session. The topographical maps show the correlation coefficient (Pearson’s r) at each electrode for all five frequency bands. The highlighted parieto-occipital electrodes in the alpha band map represent the only statistically significant cluster identified by the permutation test. The scatter plot on the right visualizes this relationship, plotting the mean alpha ERSP from the significant parieto-occipital cluster against the individual *ΔHRD* values for each participant. (B) The same correlation analysis was performed for the change in *HRD* from baseline to the T2 session, which revealed a similar significant positive correlation in the alpha band over a parieto-occipital cluster. In both sessions, the positive correlation indicated that higher alpha power was associated with higher *ΔHRD* value, reflecting less psychophysiological habituation of the cardiac orienting response to emotional stimuli.

The correlation analysis revealed a significant positive correlation exclusively in the alpha band for both sessions (Fig. 5). Specifically, at T1, a stronger post-stimulus alpha power increase was significantly associated with higher changes in *HRD* (*ΔHRD*) value, reflecting impaired emotional habituation. This positive correlation was localized on a parieto-occipital cluster of electrodes (r = 0.710, *p* = .003, Figure 5A). Similarly, at T2, higher alpha power again correlated with a higher *ΔHRD* value, also reflecting less habituation, in a nearly identical parieto-occipital cluster (r = 0.618, *p* = .014, Figure 5B). No other frequency bands yielded significant correlations with *ΔHRD* at either of the follow-up sessions.

### Effects of REM sleep fragmentation on actigraphic-recorded sleep following the experimental sessions

The comparisons of sleep macrostructure from actigraphic monitoring of the two nights following the laboratory nights showed no significant differences between the CTR and FRG conditions, excluding any rebound effects of our REM sleep fragmentation procedure on subsequent sleep (see section 3.3 and Tables S7 and S8 in the Supplementary Materials for details).

## Discussion

In the present study, we investigated how fragmenting REM sleep in healthy subjects affected emotional memory and reactivity by implementing a new methodological approach that reduced sleep alterations when studying REM sleep functions. We highlighted that vibrotactile stimulation led to REM sleep fragmentation, with only little effect on sleep macrostructure. Furthermore, REM fragmentation compromised psychophysiological reactivity habituation, as indexed by a sustained HRD response to emotional stimuli across sessions, without altering subjective evaluations of the emotional events or their consolidation. We also identified a direct link between this physiological effect and the EEG correlate of stimulation during REM sleep, in which greater stimulation-induced parieto-occipital alpha power was associated with less cardiac habituation. These results provide crucial evidence for the role of REM sleep continuity in dampening psychophysiological reactivity, offering strong support for the SFSR hypothesis and the recent framework proposed by Cabrera and colleagues [27]. Thus, our findings underscore the importance of consolidated REM sleep for proper emotional processing, as recently highlighted in animals [27] and clinical populations [22–26].

Targeted vibrotactile stimulation successfully fragmented REM sleep continuity without affecting nocturnal awakenings, WASO, TST, and SE. Beyond macrostructural sleep parameters, these results were confirmed by the transition matrix, which showed that the experimental induction of cortical arousals led to REM sleep fragmentation primarily through transitions to N1, without altering the probability that wakefulness followed REM sleep. Furthermore, the analysis of the EEG correlates of wrist-applied vibrotactile stimulation revealed that the stimulation induced widespread topographical increases in the alpha, sigma, and beta bands, alongside a more transient increase in the low-gamma band, predominantly localized to centro-parieto-occipital regions. All these changes in brain activity power are consistent with the concept of arousal during sleep [43], denoting a transient shift away from sleep-like activity.

Experimentally induced REM sleep fragmentation did not impair the overnight processing of the declarative component of the emotional information. Memory performance declined over time, did so equally in both conditions, and the decline was unaffected by the emotional valence of the stimuli. This finding is consistent with our initial hypothesis, based on the recent theoretical framework proposed by Cabrera and colleagues [27]. In particular, the new perspective underscores, on the one hand, the important role of NREM sleep in consolidating the declarative component of emotional memory via replay. On the other hand, it predicts that while increasing NA levels during REM sleep (as in fragmented REM sleep) will interfere with the long-term depression plasticity responsible for the depotentiation of the affective tone and physiological habituation, it could promote and facilitate the long-term potentiation processes linked to REM sleep memory replay, possibly improving memory consolidation of the factual content [27, 32–34]. Accordingly, it is plausible that the periods of REM sleep cumulated across the night may have been sufficient to preserve memory performance [62, 63], leading to a rate of forgetting comparable to that in the CTR condition. However, while recent meta-analyses suggest that REM sleep is critical for emotional memory consolidation [1–3], these conclusions are based on a small number of studies (i.e. *n* = 8 in Schäfer) [3], prompting the authors to call for further investigation [3]. Furthermore, these studies primarily employ selective REM sleep deprivation or split-night paradigms. In contrast, our paradigm fragmented REM sleep, rather than eliminating it. Nevertheless, while our data align with our hypothesis, we cannot definitively determine whether this memory resilience is due to robust NREM-dependent consolidation mechanisms or to sufficient memory replay during the brief periods of REM sleep allowed. Regardless of which mechanism explains this resilience, our results support that REM sleep fragmentation does not specifically impair declarative memory consolidation in healthy individuals.

Experimentally induced REM sleep fragmentation did not alter resting-state HRV parameters or the subjective evaluation of emotional stimuli, although it compromised physiological adaptation to emotional stimuli. Contrasting results regarding the effect of sleep manipulation on subjective and objective indices of emotional reactivity are well documented in the literature [2]. Since distinct brain structures generate a psychophysiological or cognitive response to an emotional event [64, 65], we can assume that one night of REM sleep fragmentation compromises the functioning of subcortical structures, undermining psychophysiological habituation processes, without interfering with the functionality of the cortical structures involved in cognitively elaborated responses [26]. We reported a lack of psychophysiological habituation when examining emotional reactivity with HRD, while no effect was observed for EDA. This divergence could be explained by intrinsic differences in their susceptibility to habituation effects, which the task structure could shape. Upon stimulus onset, EDA increases in parallel with a transient HRD. However, when the same or highly similar stimuli are repeatedly presented, as in our protocol, habituation typically occurs, and its rate may differ between indices [66, 67]. Indeed, EDA tends to show a faster habituation to repeated stimuli compared with cardiac responses, also for relevant stimuli [67]. Since EDA habituation has also been shown to occur more rapidly in healthy individuals [68], our sample may have been biased toward reduced EDA sensitivity. In contrast, HRD reflects a more sensitive, complex emotional response that involves higher-order attentional and evaluative processes [69]. Although both indices represent facets of emotional reactivity, HRD’s slower habituation profile and its broader sensitivity to meaningful stimulus processing may have rendered it a more reliable indicator than EDA of the deleterious effects of REM fragmentation on psychophysiological adaptation. Thus, the absence of EDA effects in our study likely reflects a task-related habituation, without necessarily implying a functional dissociation in the emotional processes they both index.

In general, physiological habituation to previously encountered emotional stimuli demonstrated that they have been appropriately processed over time [66, 70]. Therefore, the observed lack of psychophysiological habituation indicated that REM sleep fragmentation interfered with the emotional processing during REM sleep. Our results align with those on clinical populations in which dysfunctional emotional processing due to REM sleep fragmentation has been highlighted [22, 68, 71, 72]. For instance, Wassing et al. [26] found that REM sleep fragmentation impairs the psychophysiological dampening of amygdala activity in response to known emotional events in individuals with insomnia. However, to the best of our knowledge, our study was the first to investigate REM sleep fragmentation in healthy subjects and demonstrate that experimentally inducing this condition compromised the regulatory role of REM sleep on emotional processing. To reveal this link, we employed a standardized laboratory approach rather than one assessing real-world stressors. Although this was necessary to maximize experimental control and reduce sources of variability, it does not fully replicate the richness and personal relevance of real-world emotional stressors. Therefore, a critical next step for the field will be to investigate whether these mechanisms also operate in more ecologically valid contexts.

Remarkably, our study also identified an electrophysiological correlate of the impaired habituation, highlighting alpha intrusions associated with induced cortical arousal as the potential cortical mechanism involved. Indeed, we found a direct positive relationship between the magnitude of stimulation-induced alpha power in parieto-occipital regions and the lack of HRD attenuation. The functional role of alpha oscillations is state-dependent and topographically distinct. During wakefulness, alpha power increases in task-irrelevant cortical regions and decreases in brain areas engaged in information processing, facilitating cognitive elaboration [73]. This principle appears to extend to REM sleep as well. For instance, alpha power is reduced in Broca and Wernicke areas when reported dreams are mainly characterized by expressive or receptive linguistic content [74]. However, during sleep, a critical distinction is drawn between frontal and posterior alpha oscillations. While frontal alpha is often associated with sleep-protective mechanisms [75], posterior alpha is consistently interpreted as a “wake-like” state [76]. The micro-architecture of REM sleep further differentiates alpha functions. Specifically, tonic REM is characterized by a sustained background of alpha activity and heightened responsivity to external stimuli. In contrast, phasic REM, which is thought to be dedicated to internal processing, shows a suppression of this background alpha, interrupted only by brief, momentary posterior alpha bursts [77]. Nevertheless, posterior alpha bursts in REM sleep are also reported to be independent of REM phase [76] and are indicative of a brief temporal window during which external sensory processing is restored to monitor for potential threats [76]. Clinical conditions like PTSD and insomnia, which are characterized by threat hypervigilance, are generally associated with REM sleep fragmentation due to arousal (wake-like state) intrusion in REM, interfering with emotional processing [78]. Our correlational results corroborate the distinct functional role of frontal and posterior alpha power and extend it to REM sleep. This finding offers a different perspective from a previous influential study, which proposed that the overnight dampening of amygdala reactivity was associated with frontal gamma power, suggesting that gamma activity could serve as a proxy for NA tone during REM sleep [7]. However, the assumption that REM sleep gamma directly reflects NA levels warrants careful consideration. This premise was largely based on studies in which NA manipulations were performed on awake animals, demonstrating that LC stimulation or NA microinjections could elicit gamma activity [10, 79]. It remains uncertain whether this relationship holds within the distinct neurochemical milieu of REM sleep, an uncertainty amplified by the lack of direct supporting evidence in human sleep studies. In line with this ambiguity, our data revealed no significant correlation between the stimulation-induced changes in gamma power and the modulation of the cardiac response. However, it should be noted that the ERSP induced by our stimulation paradigm in the gamma band primarily involved the lower portion of this frequency domain. Nevertheless, cortical arousals during REM sleep should induce NA bursts from the LC, thereby preventing sleep-dependent emotional reactivity adaptation to emotional stimuli [27]. Indeed, our results provide indirect support for the neurochemical principles of the SFSR hypothesis [11], assuming that the neurochemical milieu of REM sleep (characterized by low NA and high acetylcholine levels) is essential to allow emotional memory traces to disengage from the associated emotional tone. Specifically, we demonstrated that inducing cortical arousals during REM sleep is sufficient to disrupt the continuity of this sleep stage and interfere with the psychophysiological emotional reactivity dampening that occurs overnight.

Finally, a critical finding of our study is the long-term persistence of impaired habituation and the confirmation of the strong predictive role of stimulation-induced alpha activity on emotional response at the 48-h follow-up. After the laboratory sleep night, despite two subsequent nights of unperturbed sleep, participants in the FRG condition still showed no HRD attenuation. This suggests that simply reactivating the memory traces by re-exposing participants to the emotional reactivity task at T1 was insufficient to correct the maladaptive emotional processing established during the FRG night. Therefore, our data underscore a critical time window for emotional processing: undisturbed REM sleep during the first night after an emotional experience appears essential for appropriately dampening its long-term psychophysiological impact.

Notably, these results emerged after a single night of REM sleep fragmentation. While our paradigm successfully fragmented REM sleep, it represents an acute perturbation rather than the chronic disruption described in clinical populations, raising important questions for future research. To our knowledge, no human studies have investigated the chronic effects of REM sleep fragmentation on emotional reactivity. Indeed, chronic REM sleep manipulation studies in humans are scarce, typically focus on selective REM sleep deprivation rather than fragmentation, and are not directly focused on emotional reactivity [30]. Animal models, in contrast, suggest that chronic REM sleep disruption alters monoamine balance in the limbic system and can lead to anxiety-like behaviors in adulthood [80, 81]. Furthermore, prominent theoretical frameworks posit that the long-term effects of such disruption are cumulative, potentially perpetuating the emotional dysregulation associated with restless REM sleep [11, 27, 82, 83]. It is plausible that a longitudinal protocol involving repeated nights of REM sleep fragmentation in healthy individuals could exacerbate these effects, potentially leading to impairments across a broader range of psychophysiological measures and even affecting subjective emotional ratings. Furthermore, since emotional memory alterations are often reported in clinical populations [84–86], despite the presence of REM sleep, a chronic fragmentation protocol may also evaluate if maladaptive emotional memory consolidation could be a consequence of chronic REM sleep disruption.

By establishing a direct link between experimentally induced REM sleep fragmentation and impaired emotional habituation, our study provides strong support for the hypothesis that REM sleep continuity is critical for emotional processing. Therefore, this finding suggests that the restless REM sleep characterizing different clinical populations is directly involved in the emotional dysregulation distinctive of these conditions. Our study highlights the need for further research on the link between REM sleep fragmentation and emotional health.

## Limitations

Several limitations of our study should be acknowledged.

First, we did not assess trauma exposure or PTSD symptoms. However, given the high symptomatic overlap between PTSD and the screened psychological variables, it is plausible that our inclusion criteria excluded individuals with clinically significant trauma-related psychopathology. In addition, we selected a limited convenience sample of young university students, which may restrict the generalizability of the findings. However, we strengthened our result by adopting a within-subject design and monitoring participants’ sleep schedules via actigraphy before and after the laboratory sleep nights. This monitoring confirmed adherence to regular sleep patterns during both conditions, reducing the likelihood that confounding variables biased our results. Unlike Wassing and colleagues [26] who adopted an ecological emotional reactivity task, we utilized standardized IAPS images. This reliance on a standardized laboratory paradigm, rather than personally relevant events, limits the mundane realism of our findings. However, this choice was dictated by the methodological requirements for obtaining high-quality physiological recordings. Therefore, future research incorporating assessments of personally relevant events is needed.

Moreover, regarding the emotional memory task, we opted for a recognition paradigm over a potentially more sensitive memory recall task. This decision was deliberate, as our intent was to minimize the re-elaboration of the memory trace during follow-up assessments. A recall task, by requiring active retrieval, would have constituted a new learning event at each session. This subsequent re-elaboration would have acted as a confounding factor, making it difficult to isolate the specific long-term effects of the initial overnight consolidation. Similarly, the fixed task order (emotional reactivity task preceding emotional memory task), while necessary to ensure the reliability of the psychophysiological measures, introduces the possibility of residual effects on emotional memory performance. Although we implemented an intervening distractor task and used categorically distinct stimulus sets to minimize interference, we cannot completely exclude this possibility.

In addition, we must acknowledge a limitation in our handling of EOG artifacts. We opted to apply a 5 Hz high-pass filter to remove spectral contamination from eye movements, rather than using an EOG correction. As our hypotheses concerned higher frequencies, this filtering approach preserved the integrity of our bands of interest and avoided potential signal distortions introduced by other correction methods [50, 87, 88]. However, this decision consequently precluded any analysis of the delta band. Another methodological consideration concerns the baseline period used for the ERSP analysis. While our chosen baseline window falls within the stimulation pause, avoiding direct contamination from active stimulation, we cannot entirely exclude the possibility that subtle, visibly undetectable EEG alterations induced by preceding (ineffective) stimuli within a sequence might have affected the baseline. Nevertheless, given the arousal feature (an abrupt shift toward higher frequencies), it is unlikely that minor, undetectable baseline fluctuations could have affected our results.

Finally, while our experimental manipulation successfully fragmented REM sleep, it also reduced total REM sleep time. This raises the possibility that the observed effects on emotional habituation may be attributed to REM sleep loss rather than the fragmentation process itself. However, we argue that the fragmentation process is primarily involved in our findings for two reasons. First, our key finding was a direct correlation between an EEG signature of the arousal itself (stimulation-induced parieto-occipital alpha power) and the impairment in habituation. Second, both REM duration and REM % did not correlate with ∆HRD at both T1 (REM duration, r = -0.169, *p* = .548; REM %, r = -0.150, *p* = .593) and T2 (REM duration, r = -0.142, *p* = .614; REM %, r = -0.031, *p* = .914) sessions. This supports that the disruptive process of fragmentation, not merely the loss of REM time, is the critical mechanism underlying our results. This finding offers a data-driven contribution to the ongoing debate on whether the effects of REM sleep disruption stem from fragmentation rather than an overall reduction [30]. While our design does not permit a direct comparison with total REM suppression, it provides a model that mitigates major confounds (e.g. stress), often associated with complete deprivation methods. Nevertheless, future research should aim to further disentangle these factors.

## Conclusion

We demonstrated that fragmenting REM sleep on the first night after an emotional experience impairs subsequent psychophysiological habituation, without affecting emotional memory consolidation and its subjective ratings. The absence of psychophysiological habituation following REM sleep fragmentation was directly linked to a specific neural signature, characterized by an increase in cortical alpha power on posterior cortical regions induced by vibrotactile stimulation. Notably, this disruption appeared to be long-lasting, as two subsequent nights of undisrupted sleep did not restore typical emotional habituation. These results advance our understanding of the role of REM sleep in emotion regulation, supporting the view that restless REM sleep is a sleep alteration directly involved in the emotional dysregulation seen in clinical populations like insomnia and PTSD. A critical next step for the field will be to investigate whether these same mechanisms are at play in more ecologically valid contexts and in response to real-world emotional stressors. Finally, the present study validates experimentally induced REM sleep fragmentation as a powerful methodological approach to investigate REM sleep functions by means of cortical arousals.

## Supplementary Material

Supplementary_SLEEP-2025-0787_R1_zsaf409

## Data Availability

The data that support the findings of this study are available from the corresponding author upon reasonable request.
