# Supplementary material for: Experimentally induced REM sleep fragmentation affects psychophysiological habituation to emotional stimuli
Source: Sleep. 2025 Dec 23;49(4):zsaf409. doi: 10.1093/sleep/zsaf409 (PMC13089512; doi:10.1093/sleep/zsaf409)
Supplement: Supplementary_SLEEP-2025-0787_R1_zsaf409 [file supplementary_sleep-2025-0787_r1_zsaf409.docx]

**Experimentally induced REM sleep fragmentation affects psychophysiological habituation to emotional stimuli**

Lorenzo Viselli^1^, Federico Salfi^1^, Federica Naccarato^1^, Benedetto Arnone^1^, Domenico Corigliano^1,2^, Giulia Amicucci^1^, Fabiana Festucci^1^, Costanza Colombo^3^, Nicola Cellini^3^, Daniela Tempesta^1^, Michele Ferrara^1^*, Aurora D'Atri^1^*

^1^ Department of Biotechnological and Applied Clinical Sciences, University of L’Aquila, L’Aquila, Italy

^2^ Department of Psychology, Sapienza University of Rome, Rome, Italy

^3^ Department of General Psychology, University of Padova, Padova, Italy

* Corresponding author.

Prof. Michele Ferrara, *Ph.D.*

Department of Biotechnological and Applied Clinical Sciences

University of L'Aquila

Via Vetoio

67100 L’Aquila (AQ)

Italy

[michele.ferrara@univaq.it](mailto:michele.ferrara@univaq.it)

Prof. Aurora D’Atri, *Ph.D.*

Department of Biotechnological and Applied Clinical Sciences

University of L'Aquila

Via Vetoio

67100 L’Aquila (AQ)

Italy

[aurora.datri@univaq.it](mailto:aurora.datri@univaq.it)

**Supplementary Material**

**Table of Contents**

[1. Emotional reactivity task: stimulus validation and balancing 3](#_Toc214374294)

[2. Emotional memory task: stimulus database and blocks balancing 5](#_Toc214374295)

[3. Actigraphic data for sleep monitoring at home 9](#_Toc214374296)

[3.1 Data acquisition and Pre-processing 9](#_Toc214374297)

[3.2 Statistical analyses 9](#_Toc214374298)

[3.3 Results 10](#_Toc214374299)

[4. Resting state ECG recording before and after the emotional reactivity task 11](#_Toc214374300)

[4.1 Pre-processing 11](#_Toc214374301)

[4.2 Statistical analyses 12](#_Toc214374302)

[4.3 Results 13](#_Toc214374303)

[5. Effects of REM sleep fragmentation on emotional memory 14](#_Toc214374304)

[6. Effects of REM sleep fragmentation on emotional reactivity habituation 16](#_Toc214374305)

[References 19](#_Toc214374306)

# 1. Emotional reactivity task: stimulus validation and balancing

To build the emotional reactivity task, we selected 56 images (28 negative and 28 neutral). The negative images were sourced from the International Affective Picture System (IAPS) [1] and the neutral ones from the Mnemonic Similarity Task (MST) [2]. Because the MST database lacked normative valence and arousal ratings, we first conducted a validation study to obtain these ratings. For this purpose, 25 university students (mean age ± SD, 24.68 ± 2.40, 14 males) completed an online picture evaluation task via the Pavlovia software (Open Science Tools, Nottingham, UK). This task requested to provide valence and arousal ratings for the entire pool of selected images using the Self-Assessment Manikin (SAM) [3], on a 1-to-9 Likert scale. Subsequently, this total pool of images was divided into four smaller, numerically identical blocks: two blocks of negative images (E1_Neg, E2_Neg) and two blocks of neutral images (E1_Neu, E2_Neu). Within each emotional category, these blocks were balanced for their mean arousal and valence scores, using the normative values obtained from our validation study. Independent samples t-tests (Students’ t) confirmed that there were no significant differences between the blocks within the same emotional category (all p ≥ 0.780, Table S1).

**Table S1.** Mean ± SD of valence and arousal values for each block of images of the emotional reactivity task and their statistical comparisons.

|  |  |  |  |  | Valence | | Arousal | |
| --- | --- | --- | --- | --- | --- | --- | --- | --- |
| Block | Valence | Arousal | Comparison | | t_26_ | p | t_26_ | p |
| E1_Neg | 2.15 ± 0.39 | 6.36 ± 0.50 | E1_Neg - E2_Neg | | -0.02 | 0.987 | 0.04 | 0.965 |
| E2_Neg | 2.15 ± 0.49 | 6.36 ± 0.52 |  |  |  |  |  |  |
| E1_Neu | 5.72 ± 0.25 | 2.48 ± 0.34 | E1_Neu - E2_Neu | | -0.28 | 0.780 | -0.15 | 0.878 |
| E2_Neu | 5.74 ± 0.22 | 2.50 ± 0.34 |  |  |  |  |  |  |

These pre-balanced blocks were then combined to create four distinct versions of the task (Version 1.1 = E1_Neg + E1_Neu; Version 2.2 = E2_Neg + E2_Neu; Version 1.2 = E1_Neg + E2_Neu; Version 2.1 = E2_Neg + E1_Neu). This structured design was chosen over pure randomisation to ensure that the stimulus properties were counterbalanced across participants and conditions, preventing potential confounds (e.g., one condition accidentally containing more arousing images than another). The properties of each stimulus are reported in Table S2.

**Table S2.** Means valence and arousal ratings for negative and neutral stimuli of the emotional reactivity task and their ID.

| Negative images (IAPS) | | | Neutral images (MST) | | |
| --- | --- | --- | --- | --- | --- |
| ID | Val-M | Aro-M | ID | Val-M | Aro-M |
| 3000 | 1.48 | 7.48 | Set 6 025a | 5.88 | 3.04 |
| 3061 | 1.80 | 6.84 | Set 6 161a | 5.40 | 2.76 |
| 3069 | 1.64 | 6.80 | Set 2 014a | 5.96 | 2.72 |
| 3071 | 2.04 | 6.56 | Set 3 188b | 6.04 | 2.68 |
| 3170 | 2.04 | 6.60 | Set 3 176a | 5.64 | 2.64 |
| 3266 | 2.16 | 6.64 | Set 3 087a | 6.12 | 2.64 |
| 3015 | 2.08 | 6.52 | Set 3 020b | 5.44 | 2.60 |
| 3051 | 2.20 | 6.16 | Set 1 076b | 5.76 | 2.60 |
| 3064 | 2.12 | 6.12 | Set 2 023a | 5.96 | 2.56 |
| 3080 | 2.36 | 6.04 | Set 2 062a | 5.88 | 2.40 |
| 3131 | 3.12 | 6.00 | Set 2 185b | 5.48 | 2.24 |
| 3213 | 2.44 | 5.84 | Set 4 182b | 5.40 | 2.04 |
| 3261 | 2.28 | 5.96 | Set 5 125a | 5.60 | 2.04 |
| 3400 | 2.32 | 5.56 | Set 6 039b | 5.48 | 1.80 |
| 3016 | 1.36 | 7.36 | Set 3 116a | 5.24 | 3.00 |
| 3019 | 1.36 | 7.08 | Set 3 053a | 6.00 | 3.00 |
| 3053 | 1.64 | 6.76 | Set 3 030b | 5.76 | 2.68 |
| 3062 | 1.80 | 6.68 | Set 1 088b | 5.84 | 2.68 |
| 3063 | 2.16 | 6.60 | Set 1 090b | 5.72 | 2.64 |
| 3068 | 2.16 | 6.48 | Set 1 108b | 5.84 | 2.64 |
| 3100 | 2.16 | 6.44 | Set 2 118b | 5.64 | 2.60 |
| 3150 | 2.52 | 6.36 | Set 2 168b | 5.76 | 2.56 |
| 3060 | 2.48 | 6.12 | Set 2 178a | 5.92 | 2.52 |
| 3102 | 1.92 | 6.04 | Set 4 060a | 5.96 | 2.52 |
| 3120 | 2.56 | 5.92 | Set 4 055a | 5.60 | 2.20 |
| 3130 | 2.36 | 5.92 | Set D 137b | 6.00 | 2.16 |
| 3140 | 2.56 | 5.64 | Set 4 181a | 5.76 | 2.04 |
| 9405 | 3.08 | 5.60 | Set 5 143b | 5.36 | 1.80 |

# 2. Emotional memory task: stimulus database and blocks balancing

To construct the emotional memory task, a total pool of 480 images (240 negative, 240 neutral) was selected from the IAPS and Nencki Affective Picture System (NAPS) databases [1,4]. This pool was first divided into eight blocks of 60 stimuli each (four negative: S1_Neg to S4_Neg; and four neutral: S1_Neu to S4_Neu). Each block was then further subdivided into three sub-blocks of 20 stimuli each (e.g., S1A, S1B, S1C). A balancing procedure was implemented. Using the standardised valence and arousal values from the original databases, we ensured that all blocks and sub-blocks were perfectly balanced within each emotional category. A series of ANOVAs, with block number as the fixed factor, confirmed no significant differences in valence or arousal between any of the blocks or sub-blocks (all Bonferroni corrected p > 0.999). The mean properties for each block are detailed in Table S3.

**TABLE S3.** Mean ± SD of valence and arousal value for each block of images in the emotional memory task.

| Block | Valence | Arousal |
| --- | --- | --- |
| S1_Neg | 2.50 ± 0.40 | 6.28 ± 0.58 |
| S2_Neg | 2.49 ± 0.37 | 6.28 ± 0.71 |
| S3_Neg | 2.50 ± 0.37 | 6.29 ± 0.59 |
| S4_Neg | 2.50 ± 0.34 | 6.29 ± 0.62 |
| S1_Neu | 5.02 ± 0.31 | 4.03 ± 0.87 |
| S2_Neu | 5.02 ± 0.26 | 4.03 ± 0.92 |
| S3_Neu | 5.02 ± 0.25 | 4.02 ± 0.90 |
| S4_Neu | 5.02 ± 0.25 | 4.03 ± 0.86 |

These pre-balanced blocks served as the basis for a counterbalancing scheme to avoid item-specific effects across sessions and conditions. For the encoding phase, four sets of 120 stimuli were created by combining corresponding negative and neutral macro-blocks (e.g., S1_Neg + S1_Neu). For the recognition phases, a given sub-block of "OLD" images (seen during encoding) was intermixed with "NEW" distractor sub-blocks drawn from different blocks across the three test sessions. This design ensured that stimulus exposure, novelty, and emotional properties were controlled across the entire experiment. The properties of each stimulus are reported in Table S4.

**Table S4.** Mean valence and arousal ratings for negative and neutral stimuli of the emotional memory task and their ID.

| Negative images | | | | | | Neutral images | | | | | |
| --- | --- | --- | --- | --- | --- | --- | --- | --- | --- | --- | --- |
| IAPS | | | NAPS | | | IAPS | | | NAPS | | |
| ID | Val-M | Aro-M | ID | Val-M | Aro-M | ID | Val-M | Aro-M | ID | Val-M | Aro-M |
| 2710 | 2.52 | 5.46 | A063h | 2.47 | 7.06 | 7006 | 4.88 | 2.33 | P104h | 5.38 | 4.12 |
| 3005.1 | 1.63 | 6.20 | A060h | 3.02 | 6.59 | 7950 | 4.94 | 2.28 | O314h | 4.93 | 4.70 |
| 6838 | 2.45 | 5.80 | F284h | 2.43 | 6.83 | 7179 | 5.06 | 2.88 | O313h | 5.46 | 4.41 |
| 6530 | 2.76 | 6.18 | F010h | 2.24 | 7.73 | 7044 | 4.69 | 3.94 | O224h | 4.89 | 4.91 |
| 6210 | 2.95 | 6.34 | L139h | 2.43 | 6.44 | 7595 | 4.55 | 3.77 | O179h | 4.85 | 4.98 |
| 8485 | 2.73 | 6.46 | O125h | 2.02 | 6.20 | 7248 | 5.22 | 4.22 | O245h | 5.20 | 4.88 |
| 9006 | 2.34 | 5.76 | P082h | 3.00 | 5.76 | 7018 | 4.81 | 3.91 | L020h | 4.65 | 5.02 |
| 9254 | 2.03 | 6.04 | P242v | 2.86 | 7.04 | 5740 | 5.21 | 2.59 | L084v | 5.40 | 4.69 |
| 9302 | 2.32 | 5.58 | P001h | 2.69 | 6.84 | 2396 | 4.91 | 3.34 | F218h | 4.96 | 4.85 |
| 9424 | 2.87 | 5.78 | A037h | 3.03 | 6.69 | 2749 | 5.04 | 3.76 | F055h | 5.42 | 4.90 |
| 9560 | 2.12 | 5.50 | A067h | 2.96 | 6.00 | 7247 | 5.05 | 4.14 | P159h | 5.44 | 4.43 |
| 2730 | 2.45 | 6.80 | F365v | 1.98 | 7.10 | 7830 | 5.26 | 4.08 | O161v | 5.44 | 4.48 |
| 2375.1 | 2.20 | 4.88 | F007h | 2.87 | 6.65 | 7017 | 5.18 | 3.12 | O146h | 4.48 | 4.88 |
| 6200 | 2.71 | 6.21 | O002h | 2.66 | 6.27 | 7000 | 5.00 | 2.42 | O089h | 5.09 | 4.93 |
| 6350 | 1.90 | 7.29 | O149h | 2.28 | 7.18 | 7632 | 5.22 | 4.78 | O234h | 4.43 | 4.97 |
| 6834 | 2.91 | 6.28 | P118h | 2.48 | 5.98 | 7705 | 4.77 | 2.65 | O282h | 4.36 | 5.09 |
| 9400 | 2.50 | 5.99 | P086h | 3.06 | 7.02 | 7011 | 4.52 | 3.81 | L093h | 5.41 | 4.56 |
| 9570 | 1.68 | 6.14 | P136h | 2.60 | 6.10 | 2411 | 5.07 | 2.86 | L111h | 5.48 | 4.13 |
| 9425 | 2.67 | 5.92 | A057h | 2.92 | 5.69 | 5534 | 4.84 | 3.14 | L044h | 5.13 | 4.46 |
| 9321 | 2.81 | 6.24 | A068h | 2.56 | 6.63 | 2393 | 4.87 | 2.93 | F306v | 5.41 | 4.80 |
| 9040 | 1.67 | 5.82 | A064v | 2.96 | 5.98 | 7043 | 5.17 | 3.68 | O296h | 5.15 | 4.98 |
| 9007 | 2.49 | 5.03 | F041h | 3.02 | 6.69 | 7920 | 4.51 | 3.87 | O276h | 5.41 | 4.55 |
| 2800 | 1.78 | 5.49 | L002h | 2.80 | 6.44 | 7062 | 5.27 | 3.40 | O247h | 5.17 | 4.81 |
| 3350 | 1.88 | 5.72 | O285h | 2.73 | 6.53 | 7031 | 4.52 | 2.03 | O108v | 4.83 | 5.00 |
| 6550 | 2.73 | 7.09 | P233h | 2.47 | 6.92 | 7547 | 5.21 | 3.18 | O246h | 4.90 | 4.73 |
| 6831 | 2.59 | 5.55 | P235h | 2.67 | 6.57 | 7186 | 4.63 | 3.60 | L057h | 5.48 | 5.25 |
| 6540 | 2.19 | 6.83 | P003h | 2.60 | 7.11 | 7100 | 5.24 | 2.89 | L056h | 4.82 | 4.77 |
| 9630 | 2.96 | 6.06 | A033h | 2.41 | 6.61 | 6150 | 5.08 | 3.22 | F276h | 5.48 | 4.90 |
| 9185 | 1.97 | 5.65 | A056h | 1.86 | 7.50 | 2410 | 4.62 | 4.13 | A093h | 5.38 | 4.72 |
| 9250 | 2.57 | 6.60 | F016h | 2.92 | 7.02 | 2026 | 4.82 | 3.40 | P149h | 5.49 | 4.21 |
| 9620 | 2.70 | 6.11 | F302h | 3.02 | 6.13 | 2383 | 4.72 | 3.41 | O152v | 5.19 | 5.10 |
| 9075 | 1.66 | 6.04 | O001h | 2.72 | 7.15 | 9210 | 4.53 | 3.08 | O187h | 4.73 | 4.96 |
| 9300 | 2.26 | 6.00 | O157h | 2.93 | 6.04 | 7040 | 4.69 | 2.69 | O168v | 5.51 | 5.00 |
| 2900 | 2.45 | 5.09 | P225h | 2.50 | 6.82 | 7249 | 5.24 | 3.97 | O191v | 5.23 | 4.63 |
| 3220 | 2.49 | 5.52 | P004h | 2.85 | 6.49 | 7038 | 4.82 | 3.01 | L159h | 4.70 | 4.88 |
| 6242 | 2.69 | 5.43 | P002v | 2.96 | 6.40 | 7004 | 5.04 | 2.00 | L091h | 5.16 | 4.57 |
| 6571 | 2.85 | 5.59 | A013h | 2.90 | 6.73 | 7211 | 4.81 | 4.20 | L024v | 4.80 | 4.36 |
| 6213 | 2.91 | 5.86 | A032h | 2.96 | 6.30 | 7491 | 4.82 | 2.39 | F201v | 5.30 | 4.78 |
| 9412 | 1.83 | 6.72 | F293h | 2.44 | 7.08 | 1675 | 5.24 | 4.37 | F309h | 5.25 | 4.84 |
| 9940 | 1.62 | 7.15 | F143v | 1.85 | 7.44 | 2595 | 4.88 | 3.71 | P146h | 4.94 | 4.90 |
| 9927 | 2.71 | 5.29 | O144h | 3.00 | 5.68 | 2484 | 5.00 | 3.75 | O306h | 5.41 | 4.78 |
| 9163 | 2.10 | 6.53 | O003h | 2.39 | 6.79 | 9260 | 4.63 | 3.45 | O299h | 5.08 | 4.92 |
| 9252 | 1.98 | 6.64 | P226h | 1.81 | 7.40 | 7242 | 5.28 | 3.83 | O244h | 5.07 | 4.98 |
| 9571 | 1.96 | 5.64 | P008h | 2.72 | 6.36 | 7590 | 4.75 | 3.80 | O134h | 4.32 | 4.84 |
| 2703 | 1.91 | 5.78 | P039v | 2.76 | 6.49 | 7020 | 4.97 | 2.17 | O162h | 5.44 | 4.98 |
| 2717 | 2.58 | 5.70 | A027h | 2.55 | 6.34 | 7003 | 5.00 | 3.07 | L016h | 5.13 | 4.09 |
| 6830 | 2.82 | 6.21 | A071h | 2.35 | 6.68 | 7217 | 4.82 | 2.43 | L060h | 5.21 | 4.27 |
| 6510 | 2.46 | 6.96 | A008v | 2.63 | 6.80 | 7550 | 5.27 | 3.95 | F198h | 4.72 | 4.91 |
| 8230 | 2.95 | 5.91 | F290h | 2.77 | 6.27 | 2487 | 5.20 | 4.05 | F192h | 5.43 | 4.78 |
| 9905 | 2.55 | 5.93 | L007h | 2.93 | 6.33 | 2215 | 4.63 | 3.38 | O115h | 4.74 | 5.04 |
| 9050 | 2.43 | 6.36 | O283h | 2.62 | 6.89 | 2102 | 5.16 | 3.03 | O112h | 4.97 | 4.53 |
| 9491 | 2.78 | 5.69 | P127h | 2.15 | 7.62 | 7235 | 4.96 | 2.83 | O311h | 5.04 | 4.98 |
| 9295 | 2.39 | 5.11 | P038h | 1.73 | 8.05 | 7497 | 5.19 | 4.97 | O211h | 5.09 | 4.87 |
| 9183 | 1.69 | 6.58 | P119h | 3.00 | 5.70 | 7002 | 4.97 | 3.16 | O068v | 5.24 | 4.80 |
| 9340 | 2.41 | 5.16 | A075h | 3.04 | 6.43 | 7175 | 4.87 | 1.72 | L019h | 5.18 | 4.31 |
| 2981 | 2.76 | 5.97 | A016h | 2.67 | 6.92 | 7150 | 4.72 | 2.61 | L036h | 5.20 | 4.88 |
| 2053 | 2.47 | 5.25 | A054h | 2.64 | 6.53 | 7025 | 4.63 | 2.71 | F215v | 4.67 | 4.85 |
| 6821 | 2.38 | 6.29 | F018h | 2.41 | 6.80 | 7021 | 5.21 | 4.17 | A129h | 5.22 | 5.02 |
| 6300 | 2.59 | 6.61 | L118v | 3.08 | 6.69 | 5535 | 4.81 | 4.11 | P150h | 5.21 | 4.64 |
| 6230 | 2.37 | 7.35 | O139h | 2.08 | 6.64 | 2745.1 | 5.31 | 3.26 | O310h | 5.28 | 4.66 |
| 9903 | 2.36 | 5.71 | P133h | 3.00 | 6.24 | 2308 | 5.22 | 3.82 | O308h | 5.02 | 4.83 |
| 9332 | 2.25 | 5.34 | P140h | 2.42 | 6.48 | 1616 | 5.21 | 3.95 | O130h | 4.98 | 4.69 |
| 9904 | 2.39 | 6.08 | P124h | 3.02 | 6.35 | 9468 | 4.67 | 4.68 | O279h | 5.43 | 4.88 |
| 9043 | 2.52 | 5.50 | A074h | 1.70 | 7.37 | 7077 | 5.12 | 4.61 | O204h | 5.13 | 4.63 |
| 9500 | 2.42 | 5.82 | A001h | 2.57 | 6.44 | 7019 | 5.20 | 3.36 | L067h | 4.98 | 4.76 |
| 9419 | 2.55 | 5.19 | F032h | 2.63 | 6.40 | 7010 | 4.94 | 1.76 | L015h | 4.59 | 4.94 |
| 2811 | 2.17 | 6.90 | F172h | 2.45 | 6.84 | 7185 | 4.97 | 2.64 | F220h | 4.73 | 4.80 |
| 3230 | 2.02 | 5.41 | O132h | 2.88 | 6.76 | 7160 | 5.02 | 3.07 | P097h | 5.27 | 4.80 |
| 6570 | 2.19 | 6.24 | O121v | 3.04 | 6.44 | 7255 | 5.07 | 3.36 | O071h | 4.98 | 5.06 |
| 6370 | 2.70 | 6.44 | P021h | 2.70 | 6.71 | 7180 | 4.73 | 3.43 | O312h | 5.16 | 4.89 |
| 6231 | 2.49 | 6.82 | P023h | 3.06 | 7.33 | 1350 | 5.25 | 4.37 | O196h | 5.09 | 4.96 |
| 9187 | 1.81 | 6.45 | P144h | 2.96 | 6.29 | 2214 | 5.01 | 3.46 | O222h | 4.73 | 5.02 |
| 9322 | 2.24 | 5.73 | A019h | 2.68 | 6.40 | 2850 | 5.22 | 3.00 | L081h | 5.48 | 4.90 |
| 9140 | 2.19 | 5.38 | A038h | 2.96 | 6.68 | 7037 | 4.81 | 3.71 | L077v | 4.64 | 4.65 |
| 9611 | 2.71 | 5.75 | A048h | 2.85 | 6.62 | 7016 | 4.76 | 3.40 | F311h | 5.43 | 4.76 |
| 9925 | 2.84 | 5.59 | F283h | 2.48 | 7.26 | 7030 | 4.69 | 2.99 | F312h | 5.04 | 4.63 |
| 9800 | 2.04 | 6.05 | L022h | 2.75 | 6.85 | 7161 | 4.98 | 2.98 | O172v | 5.31 | 4.86 |
| 2683 | 2.62 | 6.21 | O022h | 3.04 | 6.54 | 7184 | 4.84 | 3.66 | O210h | 4.85 | 4.63 |
| 3215 | 2.51 | 5.44 | P013v | 2.53 | 6.65 | 7009 | 4.93 | 3.01 | O277h | 4.59 | 4.98 |
| 6263 | 2.48 | 6.62 | P020h | 2.43 | 6.78 | 7170 | 5.14 | 3.21 | O208h | 5.13 | 4.92 |
| 6825 | 2.81 | 5.36 | P016h | 2.45 | 7.04 | 2514 | 5.19 | 3.50 | O251v | 4.83 | 4.74 |
| 6560 | 2.16 | 6.53 | A045h | 3.02 | 6.82 | 2397 | 4.98 | 2.77 | L013v | 4.35 | 5.07 |
| 9181 | 2.26 | 5.39 | A025h | 2.65 | 6.63 | 2702 | 5.21 | 3.92 | L078h | 4.50 | 4.86 |
| 9325 | 1.89 | 6.01 | F146h | 3.07 | 6.42 | 5531 | 5.15 | 3.69 | L063h | 5.49 | 4.63 |
| 9414 | 2.06 | 6.49 | F019h | 2.79 | 6.65 | 7207 | 5.15 | 3.57 | F216h | 4.91 | 4.85 |
| 9520 | 2.46 | 5.41 | L026h | 2.48 | 6.41 | 7187 | 5.07 | 2.30 | F194h | 5.23 | 4.90 |
| 9810 | 2.09 | 6.62 | O011h | 2.87 | 5.89 | 7090 | 5.19 | 2.61 | A105h | 5.35 | 4.77 |
| 9922 | 2.78 | 5.21 | P200h | 2.27 | 6.93 | 7050 | 4.93 | 2.75 | P091h | 5.26 | 5.04 |
| 2345.1 | 2.26 | 5.50 | P009h | 2.70 | 6.51 | 7041 | 4.99 | 2.60 | O237h | 5.50 | 4.27 |
| 2688 | 2.73 | 5.98 | P128h | 2.23 | 7.75 | 7487 | 4.92 | 4.08 | O186v | 4.55 | 4.83 |
| 6563 | 1.77 | 6.85 | A077h | 2.02 | 7.48 | 5510 | 5.15 | 2.82 | O239v | 5.00 | 4.74 |
| 6212 | 2.19 | 6.01 | A042h | 2.82 | 6.32 | 2381 | 5.25 | 3.04 | O189h | 5.07 | 4.93 |
| 7380 | 2.46 | 5.88 | F153v | 2.65 | 6.83 | 2377 | 5.19 | 3.50 | O231h | 5.21 | 4.64 |
| 9413 | 1.76 | 6.81 | F028h | 2.75 | 6.72 | 7476 | 4.99 | 4.63 | L043h | 4.80 | 5.11 |
| 9184 | 2.47 | 5.75 | O004h | 3.05 | 6.47 | 7287 | 4.77 | 3.57 | L061h | 4.98 | 4.59 |
| 9326 | 2.21 | 5.89 | P201v | 1.87 | 7.51 | 7045 | 4.97 | 3.32 | F180h | 5.27 | 4.67 |
| 9530 | 2.93 | 5.20 | P017h | 2.96 | 7.44 | 7034 | 4.95 | 3.06 | P078v | 4.98 | 5.09 |
| 9941 | 2.91 | 5.83 | P125h | 2.69 | 6.14 | 7032 | 4.82 | 3.18 | O307v | 5.07 | 4.79 |
| 9421 | 2.21 | 5.04 | P022h | 2.13 | 6.48 | 7110 | 4.55 | 2.27 | O280v | 4.82 | 5.07 |
| 2751 | 2.67 | 5.18 | A207h | 3.06 | 6.69 | 7055 | 4.90 | 3.02 | O230h | 5.32 | 5.07 |
| 3180 | 1.92 | 5.77 | A078h | 2.26 | 6.68 | 5471 | 5.21 | 3.26 | O223h | 5.20 | 4.82 |
| 6260 | 2.44 | 6.93 | A024h | 2.44 | 6.75 | 2880 | 5.18 | 2.96 | O175h | 4.43 | 5.00 |
| 6360 | 2.23 | 6.33 | F031v | 2.79 | 6.81 | 2385 | 5.20 | 3.64 | L080h | 5.46 | 4.35 |
| 6415 | 2.21 | 6.20 | F152h | 2.71 | 6.60 | 1908 | 5.28 | 4.88 | L079v | 5.09 | 4.76 |
| 9301 | 2.26 | 5.28 | O007h | 2.98 | 6.24 | 8121 | 4.63 | 4.14 | F211h | 5.48 | 5.06 |
| 9429 | 2.68 | 5.63 | P075v | 2.75 | 6.63 | 7036 | 4.88 | 3.32 | O185h | 5.18 | 5.04 |
| 9908 | 2.34 | 6.63 | P142h | 2.85 | 6.04 | 7035 | 4.98 | 2.66 | O226h | 4.98 | 5.00 |
| 9427 | 2.89 | 5.50 | P143h | 2.57 | 6.19 | 7484 | 4.99 | 4.24 | O281h | 4.56 | 4.96 |
| 9635.1 | 1.90 | 6.54 |  |  |  | 7130 | 4.77 | 3.35 | O278h | 5.48 | 4.49 |
| 9900 | 2.46 | 5.58 |  |  |  | 7012 | 4.98 | 3.00 | O176v | 5.15 | 5.00 |
| 2799 | 2.42 | 5.02 |  |  |  | 7056 | 5.07 | 3.07 | L076h | 5.06 | 4.58 |
| 6313 | 1.98 | 6.94 |  |  |  | 7080 | 5.27 | 2.32 | L012h | 4.40 | 4.62 |
| 6520 | 1.94 | 6.59 |  |  |  | 2446 | 4.70 | 3.79 | F320v | 5.06 | 4.98 |
| 6243 | 2.33 | 5.99 |  |  |  | 2191 | 5.30 | 3.61 |  |  |  |
| 7359 | 2.92 | 5.36 |  |  |  | 2038 | 5.09 | 2.94 |  |  |  |
| 9920 | 2.50 | 5.76 |  |  |  | 9700 | 4.77 | 3.21 |  |  |  |
| 9921 | 2.04 | 6.52 |  |  |  | 7059 | 4.93 | 2.73 |  |  |  |
| 9428 | 2.31 | 5.66 |  |  |  | 7182 | 5.16 | 4.02 |  |  |  |
| 9610 | 2.89 | 5.23 |  |  |  | 7233 | 5.09 | 2.77 |  |  |  |
| 9430 | 2.63 | 5.26 |  |  |  | 7058 | 5.29 | 3.98 |  |  |  |
| 9909 | 2.78 | 5.98 |  |  |  | 7365 | 5.20 | 4.13 |  |  |  |
| 2095 | 1.79 | 5.25 |  |  |  | 7014 | 5.15 | 3.25 |  |  |  |
| 3500 | 2.21 | 6.99 |  |  |  | 7053 | 5.22 | 2.95 |  |  |  |
| 6312 | 2.48 | 6.37 |  |  |  | 5532 | 5.19 | 3.79 |  |  |  |
| 6250 | 2.83 | 6.54 |  |  |  | 2890 | 4.95 | 2.95 |  |  |  |
| 6315 | 2.31 | 6.38 |  |  |  | 2309 | 4.89 | 4.33 |  |  |  |
| 9423 | 2.61 | 5.66 |  |  |  | 2279 | 4.71 | 3.74 |  |  |  |
| 9600 | 2.48 | 6.46 |  |  |  |  |  |  |  |  |  |
| 9910 | 2.06 | 6.20 |  |  |  |  |  |  |  |  |  |
| 9911 | 2.30 | 5.76 |  |  |  |  |  |  |  |  |  |
| 9901 | 2.27 | 5.70 |  |  |  |  |  |  |  |  |  |
| 9902 | 2.33 | 6.00 |  |  |  |  |  |  |  |  |  |

**Note:** We simplified the ID of the NAPS images to allow appropriate table dimensions. Then, readers could use the following abbreviations to return to the original ID: A = Animals, F = Faces, L = Landscapes, O = Objects, P = People.

# 3. Actigraphic data for sleep monitoring at home

## 3.1 Data acquisition and Pre-processing

The actigraphy (GENEActiv accelerometer – Activinsights Ltd., Kimbolton, UK) was initialised through GENEActiv software (version 3.3), setting 50 Hz as measurement frequency. Actigraphic raw data were managed via CICADA (version 0.10.4, beta, Australia) deriving the following variables: Sleep Onset Latency (*SOL*, min); *#awakenings*, denoting the total number of awakenings during the sleep period; Wake After Sleep Onset (*WASO*, min); Total Sleep Time (*TST*, min); Sleep Efficiency (*SE*, %); and *#awakenings/hour*.

## 3.2 Statistical analyses

A paired-sample t-test (Students’ t) was performed to compare actigraphic sleep parameters between the CTR and FRG conditions. We investigated potential differences between conditions on actigraphic sleep variables during the two nights preceding and following the laboratory sleep nights. Paired t-tests on actigraphic sleep variables were conducted without correction for multiple comparisons, as the goal was to verify the absence of significant differences between conditions. Not applying corrections reduced the risk of inflating Type II error, thus offering a more stringent test of equivalence.

## 3.3 Results

Actigraphic data analyses were based on 13 participants for the nights preceding the laboratory sleep nights, due to missing data resulting from recording issues of 4 subjects.

Analyses of the first night of actigraphic recording, namely two nights before the laboratory sleep night (Table S5), and the night preceding the laboratory sleep night (Table S6) confirmed equivalent sleep parameters prior to the laboratory sleep night across conditions. Moreover, sleep parameters were also similar between conditions following the laboratory sleep night (Table S7) and on the night prior to the T2 testing phase (Table S8).

Here, the data analyses were based on 15 participants, as 2 participants had missing data due to recording issues.

**Table S5.** Mean ± SD of actigraphic sleep variables in each condition during the first night of actigraphic recording, and their statistical comparisons.

| **Variable** | **FRG** | **CTR** | **t_12_** | **p** |
| --- | --- | --- | --- | --- |
| SOL | 6.08 ± 5.20 | 6.08 ± 4.77 | -0.01 | >0.999 |
| #awakening | 19.08 ± 5.65 | 18.00 ± 3.96 | 0.68 | 0.507 |
| WASO | 55.41 ± 28.36 | 62.35 ± 25.95 | -0.78 | 0.448 |
| TST | 454.20 ± 69.67 | 442.34 ± 59.44 | 0.52 | 0.611 |
| SE% | 87.33 ± 5.11 | 85.87 ± 4.21 | 0.91 | 0.382 |
| #awakening/hour | 2.22 ± 0.40 | 2.18 ± 0.57 | 0.22 | 0.831 |

**Table S6.** Mean ± SD of actigraphic sleep variables in each condition during the night preceding the laboratory sleep night, and their statistical comparisons.

| **Variable** | **FRG** | **CTR** | **t_12_** | **p** |
| --- | --- | --- | --- | --- |
| SOL | 10.46 ± 22.62 | 3.85 ± 3.41 | 1.02 | 0.327 |
| #awakening | 19.31 ± 4.89 | 18.85 ± 4.30 | 0.49 | 0.632 |
| WASO | 56.04 ± 37.01 | 53.25 ± 21.57 | 0.26 | 0.802 |
| TST | 443.88 ± 74.71 | 445.06 ± 58.15 | -0.07 | 0.948 |
| SE% | 86.60 ± 6.22 | 87.54 ± 3.94 | -0.52 | 0.612 |
| #awakening/hour | 2.33 ± 0.54 | 2.26 ± 0.43 | 0.72 | 0.484 |

**Table S7.** Mean ± SD of actigraphic sleep variables in each condition during the first night at home following the laboratory sleep night, and their statistical comparisons.

| **Variable** | **FRG** | **CTR** | **t_14_** | **p** |
| --- | --- | --- | --- | --- |
| SOL | 4.27 ± 3.22 | 11.00 ± 17.20 | -1.47 | 0.165 |
| #awakening | 18.40 ± 4.19 | 18.13 ± 2.92 | 0.22 | 0.831 |
| WASO | 53.23 ± 42.63 | 41.92 ± 21.20 | 0.87 | 0.400 |
| TST | 489.17 ± 86.01 | 465.88 ± 74.57 | 1.39 | 0.186 |
| SE% | 89.22 ± 5.94 | 89.14 ± 4.84 | 0.04 | 0.970 |
| #awakening/hour | 2.05 ± 0.37 | 2.18 ± 0.42 | -1.47 | 0.164 |

**Table S8.** Mean ± SD of actigraphic sleep variables in each condition during the night preceding the T2 testing phase, and their statistical comparisons.

| **Variable** | **FRG** | **CTR** | **t_14_** | **p** |
| --- | --- | --- | --- | --- |
| SOL | 5.60 ± 6.33 | 8.93 ± 8.15 | -1.11 | 0.285 |
| #awakening | 16.20 ± 3.47 | 17.80 ± 3.26 | -1.34 | 0.203 |
| WASO | 42.62 ± 27.92 | 49.33 ± 22.64 | -0.96 | 0.356 |
| TST | 404.78 ± 55.65 | 435.87 ± 43.02 | -1.78 | 0.097 |
| SE% | 88.65 ± 5.65 | 87.48 ± 5.31 | 0.66 | 0.520 |
| #awakening/hour | 2.17 ± 0.38 | 2.22 ± 0.45 | -0.30 | 0.768 |

# 4. Resting state ECG recording before and after the emotional reactivity task

## 4.1 Pre-processing

The emotional reactivity task included 5 minutes of resting-state ECG recording before and after performing the 28 trials. For the heart rate variability (HRV) analysis, only the final 3 minutes of each 5-minute recording segment were analysed. The initial 2 minutes of the recording were discarded, serving as an accommodation period to ensure physiological stabilisation had occurred after sensor application or task completion. While the standard recommendation for short-term HRV analysis is a 5-minute recording, a 3-minute duration is considered sufficient for a reliable assessment of Low Frequency (LF) and High Frequency (HF) power [5]. This choice represented a pragmatic decision to balance the need for valid data for this secondary analysis with the extensive time demands of the overall experimental session. We conducted this recording to assess potential differences in resting-state values between conditions before and after performing the emotional reactivity task. The ECG recording was performed using a Biosignal Explorer (Biosignalsplux, PLUX wireless biosignals S.A., Lisbon, Portugal) with a sampling rate of 1000 Hz and a resolution of 16 bits. ECG raw data were operated in Artiifac, extracting the interbeat intervals (IBIs). IBIs data were processed using the Berntson detection method to identify artefacts, and cubic spline interpolation was adopted as the correction method for artefacts. From these artefact-corrected IBIs, we extracted different HRV indices [5] (Table S9).

**Table S9.** HRV computed indices accompanied by their brief description.

| **Variable** | **Description** |
| --- | --- |
| MeanRR | R-R intervals mean time (in ms), lower values indicate a higher resting heart rate. |
| SDNN | Standard Deviation of the Normal-to-Normal intervals, representing the overall variability of the heart rate that reflects the total activity of the autonomic nervous system in both its sympathetic and parasympathetic branches. Higher values indicate better cardiovascular adaptability and health. |
| RMSSD | Root Mean Square of Successive Differences. Measure short-term beat-to-beat variability, representing the activity of the autonomic nervous system parasympathetic branch. A higher RMSSD signifies relaxed state. |
| pNN50 | Percentage of the Normal-to-Normal intervals between adjacent heartbeats that differ by more than 50 ms. It is a proxy of parasympathetic nervous system activity. |
| LF (n.u.) | Low Frequency (in normalised units) represents the relative power of the variability in the low-frequency band (0.04–0.15 Hz) and is influenced by both sympathetic and parasympathetic activity. |
| HF (n.u.) | High Frequency (in normalised units) represents the relative power of the variability in the high-frequency band (0.15–0.4 Hz) and is considered a pure marker of parasympathetic nervous system activity. |
| LF/HF | Ratio of LF to HF power. This ratio is traditionally used to represent the balance between sympathetic and parasympathetic activity. A higher ratio is often interpreted as indicating a dominance of sympathetic activity, while a lower ratio suggests a dominance of parasympathetic activity. |

## 4.2 Statistical analyses

To evaluate potential differences in resting-state HRV parameters before and after the emotional reactivity task across conditions and sessions, we performed separate LMMs for each HRV index. Each model included the fixed predictors *Condition* (FRG, CTR) and *Session* (T0, T1, T2), along with their interaction (*Condition × Session*). To account for the within-subject design and intra-individual variability, a random intercept was included for each participant.

## 4.3 Results

The LMM analyses of the HRV parameters extracted from the pre-task recording revealed no significant main effect of *Condition,* nor the interaction involving it for any of the HRV indices (Table S10). A significant main effect of the *Session* emerged only for the LF and HF HRV-parameters; however, Bonferroni-corrected post-hoc comparisons did not indicate any statistically significant differences between sessions (all p > 0.050). These results reveal that fragmenting REM sleep did not affect resting-state HRV parameters.

**Table S10.** Results of the LMMs on resting-state HRV parameters preceding the emotional reactivity task

|  | Condition | | Session | | Condition × Session | |
| --- | --- | --- | --- | --- | --- | --- |
| Variable | F | p | F | p | F | p |
| Mean RR | 0.05 | 0.818 | 0.24 | 0.787 | 0.16 | 0.850 |
| SDNN | 0.02 | 0.889 | 1.43 | 0.246 | 0.38 | 0.684 |
| RMSSD | 0.14 | 0.710 | 1.67 | 0.194 | 0.77 | 0.468 |
| pNN50 | 0.05 | 0.817 | 0.85 | 0.433 | 0.60 | 0.552 |
| LF | 3.07 | 0.083 | **3.60** | **0.032** | 0.72 | 0.492 |
| HF | 3.07 | 0.083 | **3.60** | **0.032** | 0.72 | 0.492 |
| LF/HF | 3.78 | 0.056 | 2.36 | 0.100 | 1.10 | 0.338 |

Similarly, the LMM on HRV parameters extracted from the post-task recording yielded no significant main effect of *Condition*, nor the interaction involving it for any HRV parameters (Table S11). A significant main effect of *Session* was found only for the SDNN and pNN50 HRV parameters. Post-hoc comparison indicated that SDNN values were higher at T2 compared to T0 (p = 0.013). Likewise, pNN50 was higher at T2 compared to both T0 (p = 0.022) and T1 (p = 0.017). Although interpreting SDNN from short-term recording requires caution, the concurrent increase in both SDNN and pNN50 at T2 suggests an elevated parasympathetic drive following the task in the final session. Importantly, post-task autonomic recovery was comparable between conditions.

**Table S11.** Results of the LMMs on resting-state HRV parameters following the emotional reactivity task

|  | Condition | | Session | | Condition × Session | |
| --- | --- | --- | --- | --- | --- | --- |
| Variable | F | p | F | p | F | p |
| Mean RR | 0.14 | 0.709 | 3.10 | 0.051 | 0.33 | 0.721 |
| SDNN | 0.00 | 0.966 | **4.28** | **0.017** | 1.29 | 0.281 |
| RMSSD | 0.03 | 0.858 | 2.79 | 0.068 | 0.98 | 0.381 |
| pNN50 | 0.34 | 0.562 | **5.22** | **0.007** | 0.51 | 0.605 |
| LF | 0.13 | 0.723 | 0.08 | 0.924 | 0.86 | 0.429 |
| HF | 0.13 | 0.723 | 0.08 | 0.924 | 0.86 | 0.429 |
| LF/HF | 0.19 | 0.665 | 0.43 | 0.651 | 0.77 | 0.464 |

# 5. Effects of REM sleep fragmentation on emotional memory

As reported in the main manuscript, the LMM on *d’* indicated no significant main effect of the factor *Condition* or any interaction involving it (all p ≥ 0.141). Therefore, our manipulation did not impact emotional memory performance. The only significant finding from this analysis was a main effect of the factor *Session* (F_(2,176)_ = 79.25, p < 0.001). Bonferroni-corrected planned comparisons revealed that emotional memory performance progressively declined over time. Performance decreased from baseline (T0) to the post-sleep assessment (T1) (mean difference = 0.37, t = 4.95, p < 0.001), and decreased further from T1 to the 48-hour follow-up (T2) (mean difference = 0.56, t = 7.55, p < 0.001) (Figure S1).

**
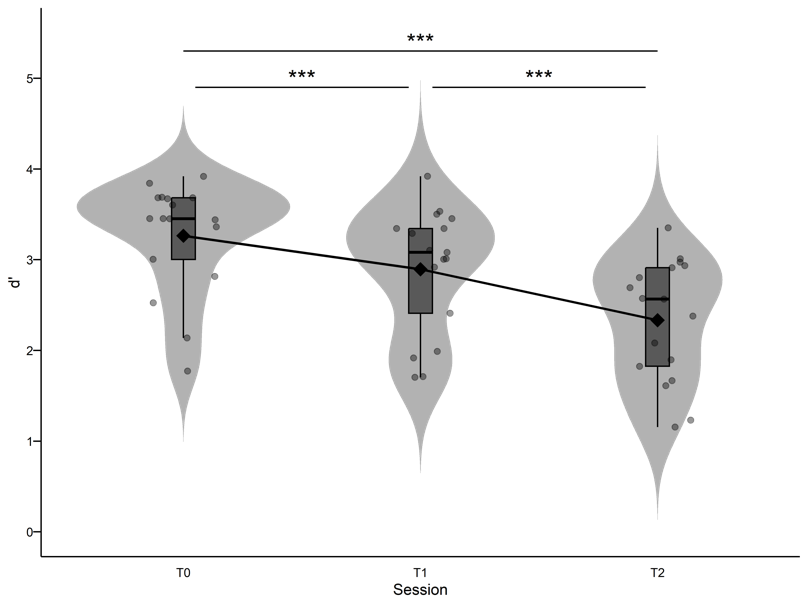
**

**Figure S1.** **Main effect of *Session* on *d’*.**

The plot illustrates the probability distribution for each session (violin plots), the median and interquartile range (boxplots), and individual participant means (dots). The line and diamonds represent the Estimated Marginal Means and their trend over time. Horizontal bars denote significant Bonferroni-corrected post-hoc comparisons. *** p < 0.001.

To provide a detailed analysis of the components underlying the *d’*, we conducted an LMM on both the *hit rate* and *false alarm rate*. Corroborating the primary *d’* analysis, neither model revealed a significant main effect of the *Condition* (all p ≥ 0.228) nor any significant interaction involving it (all p ≥ 0.120).

For the *hit rate*, the LMM indicated significant main effects of both *Session* (F_(2,176)_ = 77.20, p < 0.001) and *Stimulus Type* (F_(1,176)_ = 6.90, p = 0.009). Bonferroni-corrected post-hoc comparisons indicated that the ability to recognise OLD images as seen declined over time, with the hit rate decreasing significantly from T0 to T1 (mean difference = 0.05, t = 3.40, p = 0.002) and from T1 to T2 (mean difference = 0.14, t = 8.65, p < 0.001). The main effect of *Stimulus Type* indicated that the ability to correctly identify OLD stimuli was higher for negative stimuli than for neutral stimuli (mean difference = 0.03, t = 2.63, p = 0.009).

For the *false alarm rate*, the LMM revealed only a significant effect of the factor *Stimulus Type* (F_(1,176)_ = 8.99, p = 0.003). Bonferroni-corrected post-hoc analysis indicated that participants were more likely to erroneously identify negative NEW images as seen when they were negative compared to neutral NEW images (mean difference = 0.01, t = 3.00, p = 0.003).

For completeness, Table S12 presents descriptive statistics for *d'*, *hit rate*, and *false alarm rate*, divided by *Condition*, *Session*, and *Stimulus Type*.

**Table S12.** Mean ± SD of *d'*, *hit rate*, and *false alarm rate* partitioned by the *Condition*, *Session*, and *Stimulus Type*.

| **Condition** | **Session** | **Stimulus Type** | **d'** | **Hit rate** | **false alarm rate** |
| --- | --- | --- | --- | --- | --- |
| CTR | T0 | Neg | 3.28 ± 0.81 | 0.90 ± 0.14 | 0.05 ± 0.04 |
| FRG | T0 | Neg | 3.23 ± 0.59 | 0.91 ± 0.09 | 0.05 ± 0.05 |
| CTR | T0 | Neu | 3.36 ± 0.75 | 0.89 ± 0.16 | 0.04 ± 0.04 |
| FRG | T0 | Neu | 3.19 ± 0.60 | 0.87 ± 0.14 | 0.03 ± 0.02 |
| CTR | T1 | Neg | 3.00 ± 0.75 | 0.87 ± 0.13 | 0.05 ± 0.04 |
| FRG | T1 | Neg | 2.75 ± 0.81 | 0.83 ± 0.17 | 0.07 ± 0.06 |
| CTR | T1 | Neu | 2.92 ± 0.79 | 0.84 ± 0.15 | 0.05 ± 0.06 |
| FRG | T1 | Neu | 2.91 ± 0.79 | 0.82 ± 0.17 | 0.04 ± 0.04 |
| CTR | T2 | Neg | 2.28 ± 0.86 | 0.72 ± 0.22 | 0.07 ± 0.08 |
| FRG | T2 | Neg | 2.43 ± 0.66 | 0.73 ± 0.19 | 0.05 ± 0.04 |
| CTR | T2 | Neu | 2.41 ± 0.88 | 0.69 ± 0.20 | 0.05 ± 0.05 |
| FRG | T2 | Neu | 2.21 ± 0.73 | 0.66 ± 0.19 | 0.05 ± 0.05 |

# 6. Effects of REM sleep fragmentation on emotional reactivity habituation

As reported in the main text, the LMM performed on *Arousal* ratings revealed no significant main effects or interaction involving the factor *Condition* (all p ≥ 0.083). However, the analysis highlighted a significant main effect of the factor *Stimulus Type* (F_(1,53.97)_ = 1884.96, p < 0.001), indicating that negative pictures were perceived as more arousing than neutral pictures (mean difference = 4.65, t = 43.42, p < 0.001) (Figure S2).

**
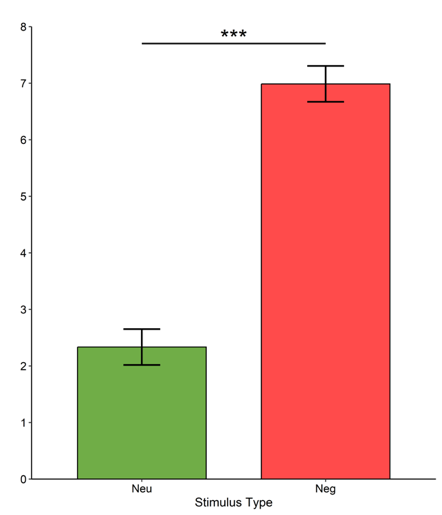
**

**Figure S2.** **The main effect of Stimulus Type for self-reported *Arousal* ratings.**

The bar chart illustrates that negative (Neg) stimuli were rated as significantly more arousing than neutral (Neu) stimuli. Error bars represent the standard error of the mean. *** p < 0.001.

Similarly, the LMM for *Valence* ratings revealed a significant *Condition × Stimulus Type* interaction (F_(1, 2791.37)_ = 9.23, p = 0.002) (Figure S3). Bonferroni-corrected post-hoc comparisons revealed that negative stimuli were rated as less pleasant than neutral stimuli in both the CTR and FRG conditions. Furthermore, neutral stimuli were rated as significantly more pleasant in the FRG condition compared to the CTR condition.


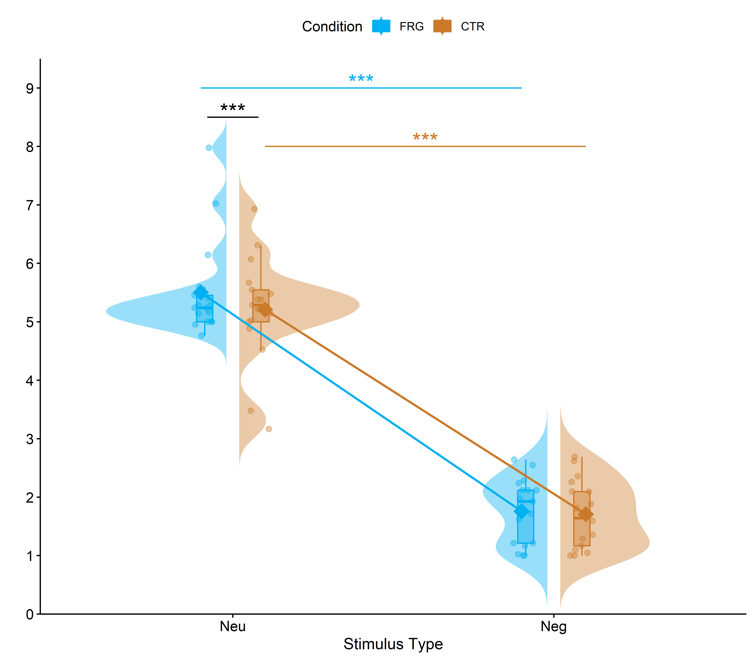


**Figure S3.** **The interaction effect between *Condition* and *Stimulus Type* on *Valence* ratings.**

The raincloud plot displays individual participant data (dots), boxplots (indicating the median and interquartile range), and violin plots (showing the data distribution) for the FRG (light blue) and CTR (copper) conditions, across neutral (neu) and negative (neg) *Stimulus Type*. Horizontal bars denote significant post-hoc comparisons: the light blue bar indicates the comparison between stimulus types within the FRG condition, the copper bar denotes the comparison between stimulus types within the CTR condition, and the black bar depicts the comparison between conditions for neutral stimuli (short bar). *** p < 0.001.

The LMM analyses carried out on *CDA.SCR* highlighted no significant effect or interaction involving the factor *Condition* (all p ≥ 0.069). The analysis revealed a significant *Session × Stimulus Type* interaction (F_(2,2014.78)_ = 14.99, p < 0.001, Figure S4). Bonferroni-corrected planned comparisons indicated that at baseline (T0), physiological arousal to negative stimuli was greater than that to neutral stimuli (mean difference = 0.01, t = 7.02, p < 0.001). This differential response disappeared in the subsequent sessions, specifically, the *CDA.SCR* magnitude for negative stimuli decreased from T0 to T1 (mean difference = 0.01, t = 7.44, p < 0.001), indicating habituation.


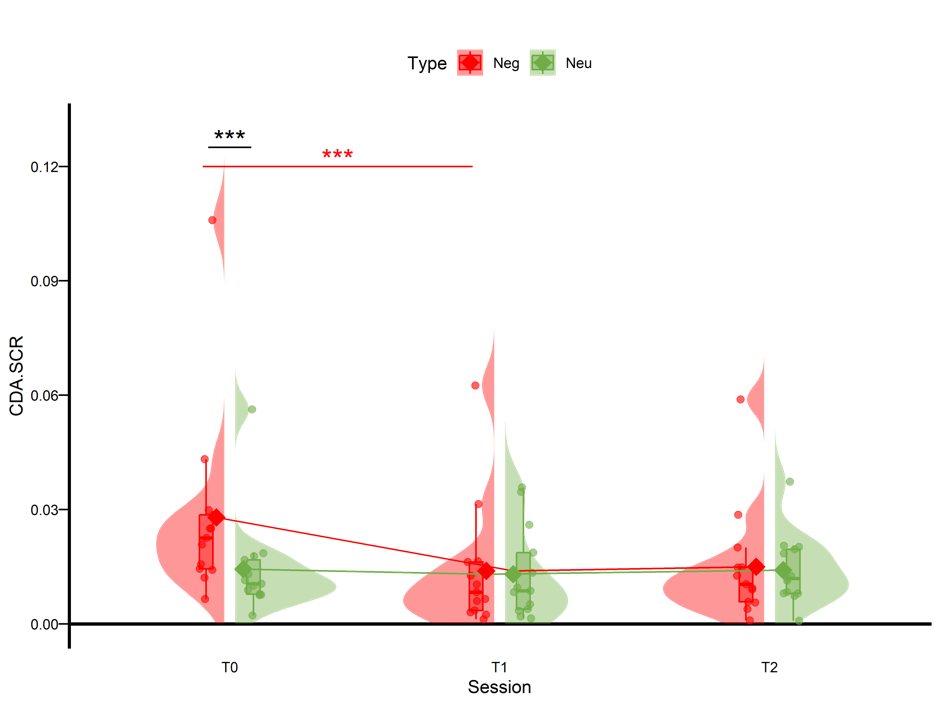


**Figure S4.** **The interaction effect between *Session* and *Stimulus Type* on *CDA.SCR* index.**

At baseline (T0), arousal was significantly greater in response to negative (red) stimuli compared to neutral (green) stimuli. This differential response disappeared in the subsequent sessions due to a significant decrease in *CDA.SCR* for negative stimuli from T0 to T1, indicating a habituation effect of the electrodermal response to negative stimuli.

The raincloud plot displays individual participant data (dots), boxplots showing the median and interquartile range, and violin plots illustrating the distribution of the data. Horizontal bars denote significant Bonferroni-corrected post-hoc comparisons: the black bar indicates a significant between-stimulus type comparison, the red bar denotes a significant comparison within the stimulus type. *** p < 0.001.

# References

1. Lang PJ, Bradley MM, Cuthbert BN. *International Affective Picture System (IAPS): Affective Ratings of Pictures and Instruction Manual*. NIMH, Center for the Study of Emotion & Attention Gainesville, FL; 2005.

2. Stark SM, Kirwan CB, Stark CEL. Mnemonic Similarity Task: A Tool for Assessing Hippocampal Integrity. *Trends Cogn Sci*. 2019;23(11):938-951. doi:10.1016/J.TICS.2019.08.003

3. Bradley MM, Lang PJ. Measuring emotion: The self-assessment manikin and the semantic differential. *J Behav Ther Exp Psychiatry*. 1994;25(1):49-59. doi:10.1016/0005-7916(94)90063-9

4. Marchewka A, Żurawski Ł, Jednoróg K, Grabowska A. The Nencki Affective Picture System (NAPS): Introduction to a novel, standardized, wide-range, high-quality, realistic picture database. *Behav Res Methods*. 2014;46(2):596-610. doi:10.3758/s13428-013-0379-1

5. Cygankiewicz I, Zareba W. Heart rate variability. In: Buijs RM, Swaab DF. *Handbook of Clinical Neurology*. Elsevier; 2013:379-393. doi:10.1016/b978-0-444-53491-0.00031-6
